# Comparative metagenomic analysis of microbial community compositions and functions in cage aquaculture and its nearby non-aquaculture environments

**DOI:** 10.3389/fmicb.2024.1398005

**Published:** 2024-05-22

**Authors:** Zetian Liu, Pandeng Wang, Jialing Li, Xiaoqing Luo, Ya Zhang, Xiaohong Huang, Xin Zhang, Wenjun Li, Qiwei Qin

**Affiliations:** ^1^Guangdong Laboratory for Lingnan Modern Agriculture, College of Marine Sciences, South China Agricultural University, Guangzhou, China; ^2^State Key Laboratory of Biocontrol, Guangdong Provincial Key Laboratory of Plant Resources and Southern Marine Science and Engineering Guangdong Laboratory (Zhuhai), School of Life Sciences, Sun Yat-sen University, Guangzhou, China; ^3^School of Marine Biology and Fisheries, Hainan University, Haikou, Hainan, China; ^4^State Key Laboratory of Desert and Oasis Ecology, Xinjiang Institute of Ecology and Geography, Chinese Academy of Sciences, Ürümqi, China; ^5^Laboratory for Marine Biology and Biotechnology, Pilot National Laboratory for Marine Science and Technology (Qingdao), Qingdao, China; ^6^Southern Marine Science and Engineering Guangdong Laboratory (Zhuhai), Zhuhai, China

**Keywords:** cage aquaculture, eutrophication, metagenome sequencing, *Vibrio*, chemical cycling

## Abstract

In the context of burgeoning global aquaculture, its environmental repercussions, particularly in marine ecosystems, have gained significant attentions. Cage aquaculture, a prominent method, has been observed to significantly influence marine environments by discharging substantial amounts of organic materials and pollutants. It is also one of the important reasons for water eutrophication. This study investigated the impacts of cage aquaculture on microbial diversity and functional potential using metagenomics. Specifically, a comparison was made of the physicochemical indicators and microbial diversity between three grouper aquaculture cage nets in Lingshui Xincun Port and three nearby non-aquaculture area surface waters. We found that compared to non-aquaculture areas, the eutrophication indicators in aquaculture environments significantly increased, and the abundances of *Vibrio* and *Pseudoalteromonas* in aquaculture environments significantly rose. Additionally, microbial functional genes related to carbon, nitrogen, and sulfur metabolisms were also found to be significantly affected by aquaculture activities. The correlation analysis between microbial populations and environmental factors revealed that the abundances of most microbial taxa showed positive correlations with dissolved inorganic nitrogen, soluble reactive phosphorus, NH_4_^+^, and negative correlations with dissolved oxygen. Overall, this study elucidated the significant impacts of aquaculture-induced eutrophication on the diversity and functions of planktonic bacterial communities.

## 1 Introduction

In recent times, alongside the elevation of living standards, there has been a shift in the public’s food preferences from mere sustenance to a focus on quality. Marine fisheries, as a vital source of premium protein for human consumption, have seen a surge in global marine fish production (Liu et al., 2017). With dwindling wild fish stocks and escalating demand for marine products, marine aquaculture has emerged as a dominant sector in animal food production, contributing to more than 17% of the worldwide output (Lidder and Sonnino, 2012). Intensive aquaculture models, particularly cage aquaculture, are recognized for their high yield and efficiency (Calado et al., 2021). Owing to the burgeoning need for aquatic products, this form of aquaculture has gained global traction (Rasheeda et al., 2017; Xie et al., 2020). As the foremost aquaculture nation, as the world’s most important aquaculture country, China faces major challenges in coastal cage farming, including spatial constraints and environmental issues (Xie et al., 2020; Zhao et al., 2020). However, the environmental repercussions of prolonged aquaculture are significant. Issues such as nitrogen accumulation, the proliferation of related metabolites, and the excessive discharge of aquaculture effluents have been implicated in the degradation of water quality and alterations in microbial community compositions (Piedrahita, 2003; Zhang et al., 2019; Dantas et al., 2020). Meanwhile，prolonged fishery activities can contribute to eutrophication, triggering the proliferation of phytoplankton and harmful algal blooms. This eutrophication has had devastating effects on aquaculture, leading to various health issues such as oxygen depletion and parasite outbreaks, particularly in enclosed or semi-enclosed bays (Troell et al., 1997, 2009; Abreu et al., 2011). Eutrophication is a process in which the nutrient content of a water body increases, leading to changes in the nutritional status of the water body within a certain range (Meyer-Reil and Köster, 2000). Marine eutrophication is mainly caused by the input of nutrients from land sources, so eutrophication generally occurs in shallow coastal seas and bays, especially in estuaries where rivers flow into the sea. Eutrophication in estuaries, bays, and coastal waters has become a prominent ecological environmental issue worldwide (Karlsson et al., 2008; Claussen et al., 2009; Håkanson and Bryhn, 2011). Eutrophication has a significant impact on the cycling of marine nutrients (C, N, P, S, etc.), water quality, biodiversity, and the overall health of the coastal marine ecosystem (Ruihua, 1999; Paerl et al., 2003). Bacteria with rapid growth rates, as important components of aquatic environments, can exhibit sensitive and rapid responses to subtle changes in the marine environment (including pollution, physicochemical properties, and biological environment) through their productivity and role in material cycling, thereby serving as indicators (Paerl et al., 2003).

Aquaculture, as a significant marine industry, contributes high-quality protein globally, a fact well-documented in scientific literature (Campbell and Pauly, 2013). Intensive research efforts focused on the impacts of these aquaculture practices on environmental microorganisms from aquaculture (Murphy and Riley, 1962; Song et al., 2009; Liu et al., 2015). A noteworthy study from 2015 at a Chinook salmon farm in New Zealand underscored the profound influence of organic enrichment on the composition of bacterial communities (Dowle et al., 2015). Further research by Zhang et al. (2019) revealed a variety of carbon fixation mechanisms, such as the Calvin–Benson–Bassham (CBB), the Wood–Ljungdahl (WL), and the 3-hydroxypropionate (3-HP) pathways, in the microbiomes of *Penaeus vannamei* in pond water, sediment, and effluent under different cultivation conditions. The study also highlighted active sulfur and phosphorus cycling processes during advanced stages of shrimp cultivation, in addition to common nitrification and sulfide oxidation activities. This research marked the first detailed examination of the microbial community’s structure, function, and ecological cycles in shrimp farming environments, with a focus on their interaction with environmental factors (Zhang et al., 2019). These findings emphasize that in aquaculture environments microorganisms play an essential role in the cycling of nutrients, transforming exogenous organisms, and balancing biogeochemical cycles of elements like carbon, nitrogen, phosphorus, and sulfur. These processes are vital for maintaining ecological balance and health (Bai et al., 2014; Md Zoqratt et al., 2018). While much of the existing research concentrates on pond and factory farming systems (Dowle et al., 2015; Zhang et al., 2019), studies on open aquaculture models, particularly those impacting the ocean, have been less frequent. Research on the eutrophication of seawater caused by aquaculture has mainly focused on the interaction between algae and nitrogen-phosphorus ratios (Song et al., 2009), with limited studies on the eutrophication of water bodies caused by aquaculture in relation to microorganisms.

For this research, the cage aquaculture region in Lingshui County, Hainan Province, was chosen as the focal study area. Samples from both cage aquaculture waters and adjacent non-aquaculture marine areas were collected for analysis. The objective was to investigate the effects of water eutrophication caused by aquaculture on microbial community compositions and functions.

## 2 Materials and methods

### 2.1 Project design and sampling pretreatment

Xincun Port is a natural lagoon in the southeast of Xincun Town, which is located in Lingshui County of Hainan Province. Geographically, it located at coordinates 18°23′-18°26’N and 109°58′ -110°03′E. It spans a length of 4 km north to south and a breadth of 6 km east to west, the port covers an area of approximately 22.3 km^2^. The entrance of Xincun Port faces west, with the narrowest point of the entrance being less than 100 m wide. The bay extends approximately 8.5 km from the entrance to the bay bottom, with no rivers flowing into the upper reaches. There is no obvious runoff inflow in the lagoon, and the main dynamic factors are wave and tide. The study area is characterized by irregular diurnal tides, with an average tidal range of 0.69 m and a maximum tidal range of 1.55 m. During a semi-diurnal tide period, the maximum tidal range at the entrance of the lagoon is 0.72 m and the maximum tidal range in the middle of the lagoon is 0.75 m. However, the high and low tides in the middle of the lagoon lag behind the entrance by about 10–20 min. The water quality in the study area meets Class II seawater standards. It is estimated that a complete exchange of the entire bay’s water would take about 1 month. The narrow entrance, coupled with a Sediment zone about 1 km long and 100 m wide outside the entrance, contributes to the slow water exchange in the entire bay area. Currently, due to the presence of numerous fish rafts for aquaculture, the seawater flow rate is reduced, further slowing down the water exchange process. The port is flanked by the Nanwan Peninsula, offering calm and sheltered conditions, making it an exceptional natural harbor with distinct advantages. Xincun Port also serves as a significant site for marine aquaculture in Hainan, notable for its absence of large freshwater river inflows, and for maintaining moderate water temperature and salinity throughout the year. The port’s waters are rich in plankton, supporting a variety of aquaculture species such as Kirin, Jiangli, pearl oysters, and various economic fish species. Due to the lack of scientific planning in the Xincun Port aquaculture area before 1996, a situation arose where aquaculture was conducted from the bay bottom to the mouth. The discharge of aquaculture wastewater and excess feed also led to water quality deterioration. Additionally, due to geographical reasons and poor seawater exchange, Xincun Port experienced two major red tides, resulting in economic losses of tens of millions (Yi et al., 2022). Thus, Xincun Port is considered an ideal area for study on the eutrophication of water bodies caused by aquaculture in relation to microorganisms.

**Figure 1:**
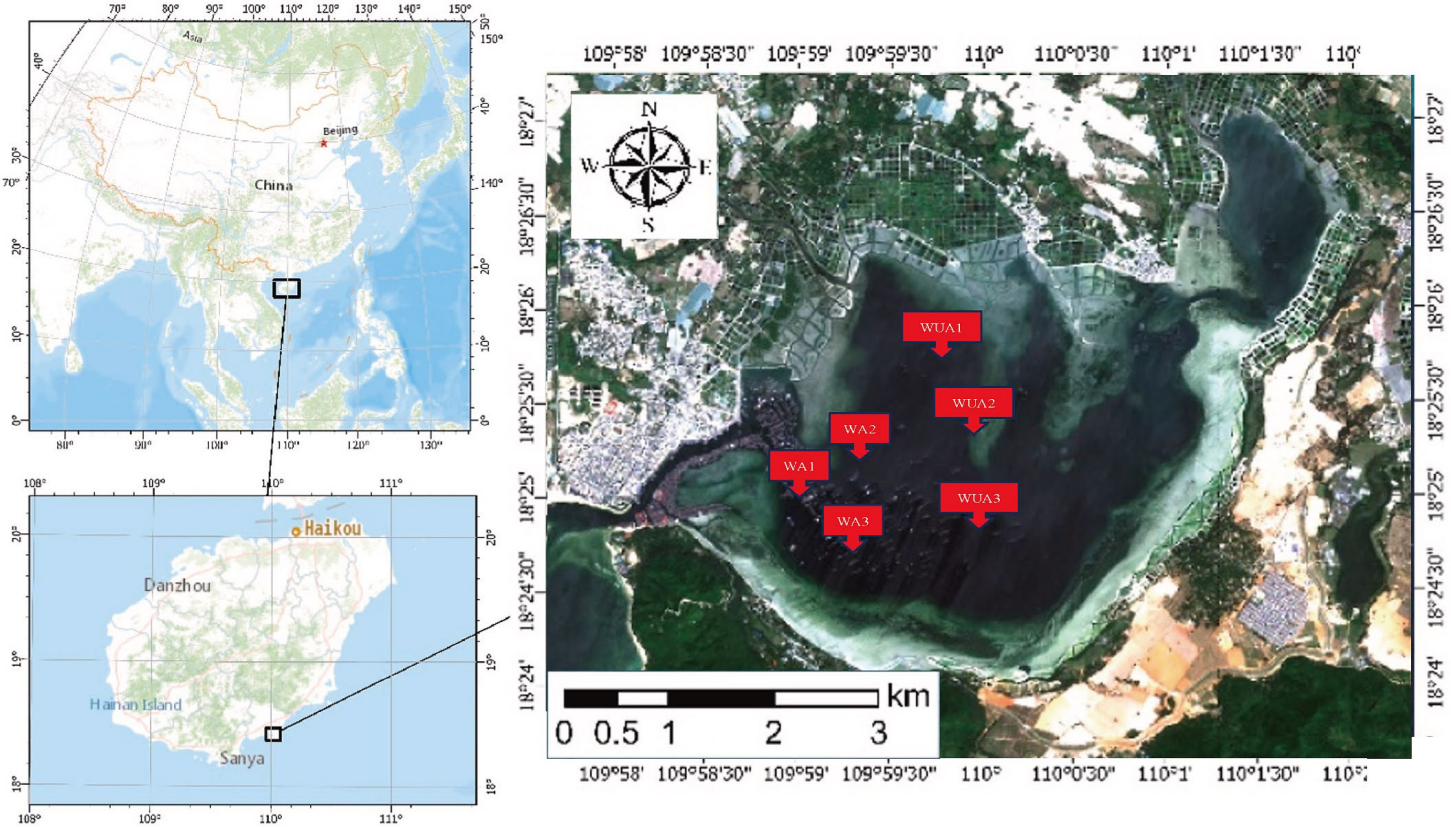
Geographical locations of the sampling sites in XinCun Port. Aquaculture area (WA1-3) Non-aquaculture area (WUA1-3).

The samples were collected from cage aquaculture and its nearby non-aquaculture areas at Xincun Port, located in Lingshui County of Hainan Province. In May 2018, for this study, surface water sampling (at a depth of approximately 1 meter) were randomly collected from three aquaculture cages and three non-aquaculture areas within the port. All sampling sites in the aquaculture areas are fixed cages, with the net body exposed above the water surface by approximately 0.7–1 m, and submerged 1.5–2.5 m underwater. The species cultivated in these areas is exclusively the Grouper (*Epinephelus lanceolatus*). The sampling sites were designated as aquaculture area 1 (WA1), aquaculture area 2 (WA2), aquaculture area 3 (WA3), non-aquaculture area 1 (WUA1), non-aquaculture area 2 (WUA2), and non-aquaculture area 3 (WUA3) (Figure 1). The physicochemical properties of the seawater samples, including water temperature, dissolved oxygen concentration, conductivity, salinity, oxidation–reduction potential and pH, were measured on site immediately after sampling, using Professional Plus handheld multiparameter meter (YSI Inc., Yellow Springs, OH). Secchi disk transparency (SD) was determined on site according to the standard protocol (Tyler, 1968). Further characterization of the seawater samples was performed in the laboratory. Portions of bulk water samples were filtered through 0.22-μm membrane filters (Merck Millipore) for quantification of dissolved nitrogen and phosphorus species. The NH_4_^+^-N, NO_3_^−^-N, and NO_2_^−^-N concentrations were determined colorimetrically as previously described (Mccrady, 1966). The dissolved inorganic nitrogen (DIN) concentration was calculated as the sum of the concentrations of these three nitrogen species. The soluble reactive phosphorus (SRP) concentration was determined using the molybdate blue method (Murphy and Riley, 1962). The result was shown in Table 1. 20-L water samples for each site were filtered through Pall Life Sciences’ 3 and 0.2 μm membranes, respectively. The membranes were stored in sterile tubes and refrigerated at −20°C for lab analysis.

**Table 1:** Description and physicochemical characteristics of the samples.

Sample ID	WA1	WA2	WA3	WUA1	WUA2	WUA3
Temperature	29.1°C	29.6°C	29.3°C	29.0°C	29.1°C	28.7°C
Latitude	18°24′48.1”N	18°25′15.3”N	18°25′16.0”N	18°24′41.1”N	18°24′54.2”N	18°25′28.9”N
Longitude	109°59′12.3″E	109°59′10.5″E	109°59′24.0″E	109°59′18.4″E	109°59′20.0″E	109°59′52.3″E
dissolved oxygen	6.31	7.13	6.22	7.43	7.59	7.37
pH	7.8	7.8	7.8	7.8	7.6	7.5
Salinity (‰)	33.57	33.61	33.58	32.73	32.78	32.91
NO_3_^−^-N (mg N/L)	0.064	0.069	0.107	0.059	0.074	0.093
NO_2_^−^-N (mg N/L)	0.014	0.004	0.017	0.005	0.004	0.019
NH_4_^+^-N (mg N/L)	0.092	0.077	0.073	0.069	0.066	0.055
Dissolved inorganic nitrogen (mg N/L)	0.892	0.7433	0.7037	0.664	0.6343	0.5252
Dissolved inorganic phosphorus (mg N/L)	0.0959	0.0779	0.0859	0.0317	0.0357	0.0558
Conductivity (μS/cm)	45,963	46,367	46,037	47,063	47,246	47,242
Oxidation–Reduction potential (mV)	45.2	46.0	46.0	41.5	40.5	45.0

### 2.2 Metagenomic sequencing and data analysis

For each sample, we extracted DNA from samples using the MoBio PowerWater DNA Isolation Kit (Qiagen), following the manufacturer’s protocol. DNA was sent to the Magigene Company for metagenomic high-throughput sequencing on the Illumina NovaSeq 6,000 PE150 platform.

Data pre-processing was executed using Readfq software (Version 8) (Chen et al., 2018). Initially, sequences with low-quality bases (Quality Value ≤38) exceeding 40 base pairs (bp) were excluded. Furthermore, sequences containing ambiguous bases (N) were also removed. Additionally, any adapter contamination extending over 15 bp was identified and eliminated. Post-cleanup, sequences were assembled into contigs with MEGAHIT (v1.0.6) (Li et al., 2015), and then evaluated using N50. The open reading frames (ORFs) were predicted using MetaGeneMark (v3.38)(Qin et al., 2010). A unigene catalog was then created with CD-HIT (v4.7) (Qin et al., 2010) and each metagenomic data was mapped to this catalog using BBMAP (Bushnell, 2014), facilitating gene abundance analysis based on read counts and gene lengths.

$GK=\frac{rk}{lk}*\frac{1}{\sum_{i=1}^{n}\frac{ri}{li}}$ (*r* was the number of reads mapped to the genes and *l* was gene s length). Unigenes were annotated by searching against NCBI’s NR database (Version: 20161115), KEGG and eggNOG using DIAMOND software (Buchfink et al., 2015).

### 2.3 Statistical analysis

All statistical analyses were performed using R v4.0.2 (R.C. Team, 2014). The analysis of key genes involved in carbon metabolism, nitrogen metabolism, and sulfur metabolism is all based on the DiTing software and their accession in the KEGG database are listed in Supplementary Tables S1–S3 (Xue et al., 2021) and the normalized relative abundance of key genes in the six samples was determined. Statistical tests of key genes involved in phosphorus and sulfur metabolism were performed using Newcombe–Wilson method test correction in STAMP (v2.0) (Parks and Beiko, 2010).

## 3 Results

### 3.1 Physicochemical characteristics of the sampling sites

We have determined through *T*-test that there are significant differences in some water quality indicators between the aquaculture area and the non-aquaculture area (Supplementary Figure S1). The concentrations of Dissolved Inorganic Nitrogen (DIN) and Soluble Reactive Phosphorus (SRP) in the aquaculture area are significantly higher than those in the non-aquaculture area. Additionally, salinity and temperature are significantly higher in the aquaculture area, while conductivity is notably higher in the non-aquaculture area. While statistically there is no significant difference in Dissolved Oxygen (DO), ammonium ions, and nitrate levels between the aquaculture and non-aquaculture areas, numerically, higher concentrations of ammonium ions and nitrate ions, along with lower levels of dissolved oxygen, are observed in the aquaculture area.

### 3.2 Metagenomic sequencing data

The metagenomic sequencing of six water samples, sourced from both aquaculture and non-aquaculture areas, yielded approximately 62.66 gigabases (Gb) of raw data. On average, each sample contributed over 10 Gb of data. From the assembly of reads, a total of 4,873,716 ORFs were identified. This led to the determination of 4,025,667 non-redundant genes. Among them, 1,073,641 genes were successfully mapped to entries in the KEGG database, as shown in Supplementary Table S4.

### 3.3 Taxonomic profiles of microbiotas in cage aquaculture and non-aquaculture environments

**Figure 2:**
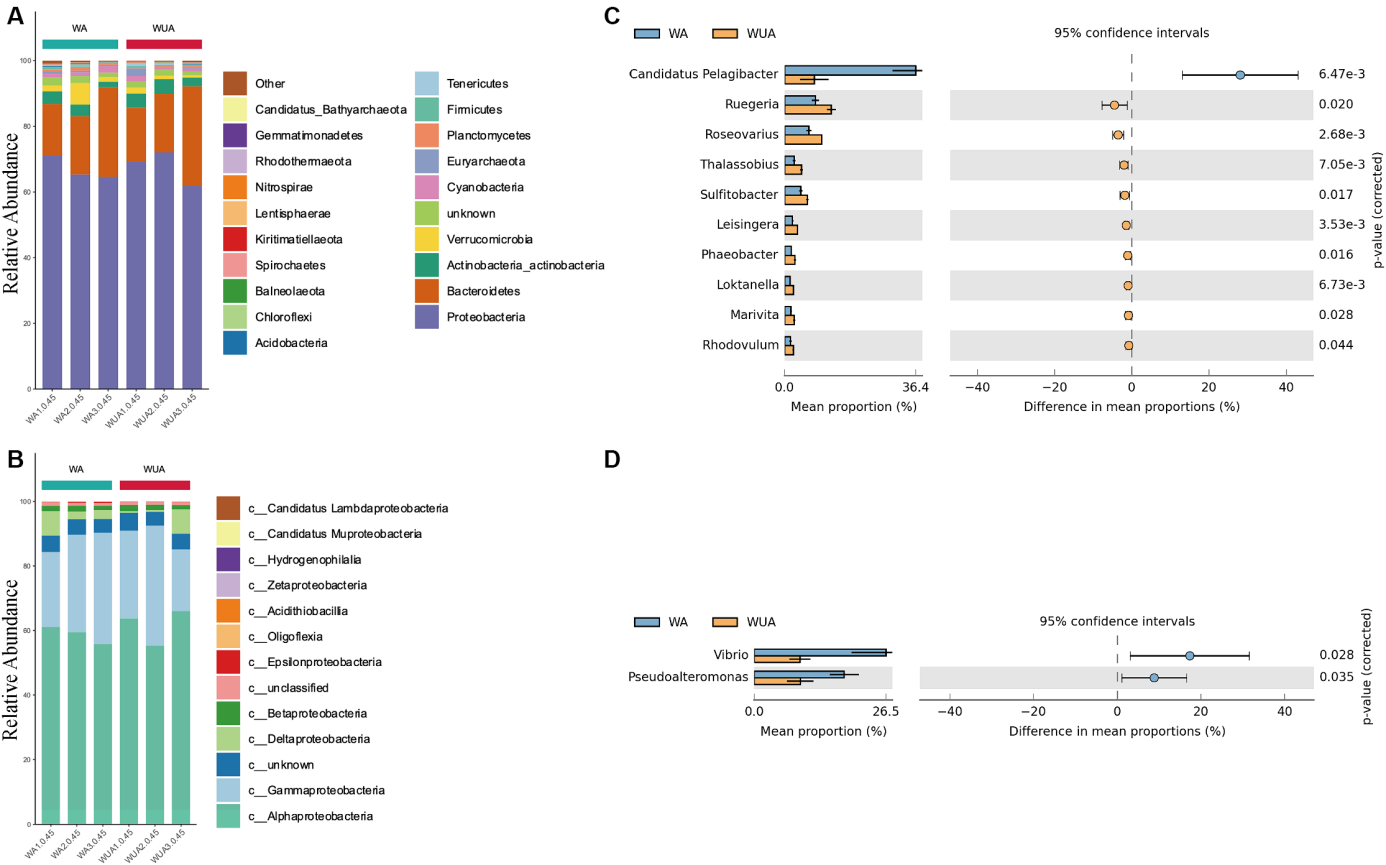
**(A)** Ten dominant phyla are shown with their relative abundance; the remaining phyla are indicated as “others.” **(B)** The relative abundance of the Proteobacteria phylum. Comparison of species abundance between aquaculture and non-aquaculture areas: **(C)** Alphaproteobacteria, **(D)** Gammaproteobacteria.

We sampled and sequenced three samples from both aquaculture and non-aquaculture areas to examine the microbial diversity in the ecosystems of aquaculture and non-aquaculture areas (Figure 2). Proteobacteria emerged as the predominant phylum, comprising approximately 65–71% of the total species in cage aquaculture samples. In non-aquaculture samples, the number is 61–72% (Figure 2A). Alphaproteobacteria and Gammaproteobacteria had dominant distribution patterns in both aquaculture and non-aquaculture areas (Figure 2B). The most abundant Alphaproteobacteria included genera *Ruegeria* (4.59–7.28%), *Roseovarius* (3.7–5.72%), *Thalassobius* (1.36–2.59%), *Sulitobacter* (2.46–3.54%), *Leisingera* (1.2–1.95%), *Phaeobacter* (1.04–1.64%), *Loktanella* (0.81–1.43%), *Marivita* (0.96–1.51%), Rhodovulum (0.87–1.37%), was higher in the WUA samples than in the WA samples. Only *Candidatus Pelagibacter* (1.58–28.03%) was higher in the WA samples than in the WUA (Figure 2C). Gammaproteobacteria, including *Vibrio* (4.34–26.08%) and *Pseudoalteromonas* (4.59–17.55%), was higher in the WA samples than in the WUA samples (Figure 2D). Given that *Vibrio* and *Pseudoalteromonas*, which are significantly enriched in the aquaculture area, may be potential pathogens. We analyzed our data for virulence genes and antimicrobial-resistant genes. We found that there were no significant differences in virulence genes between aquaculture and non-aquaculture areas (Supplementary Table S12 and Supplementary Figure S4). However, there were significant differences in antimicrobial-resistant genes between aquaculture and non-aquaculture areas, with most being enriched in the aquaculture areas (Supplementary Figure S5).

### 3.4 Distribution of key metabolic genes

To gain a more comprehensive understanding of the functional profiles of microbial communities in both aquaculture and non-aquaculture environments, our analysis specifically targeted genes encoding key enzymes linked to distinct ecosystem functions. Because less predictable is the influence of microbiota on Eutrophication of water bodies of the marine phosphorus cycle, given the patchy ecophysiological understanding of marine microbial phosphorus metabolism (Kim et al., 2021). We identified genes related to phosphorus (P) metabolism in seawater by reviewing the literature. By analyzing our data, we found some P metabolism-related genes (Supplementary Table S11). Through differential analysis, we discovered that these genes do not show significant differences between aquaculture and non-aquaculture areas (Supplementary Figure S3). So key genes for microbial carbon, nitrogen and sulfur metabolisms were searched in the metagenomes of the six water samples, and differences were observed among the two communities.

#### 3.4.1 Carbon metabolism

**Figure 3:**
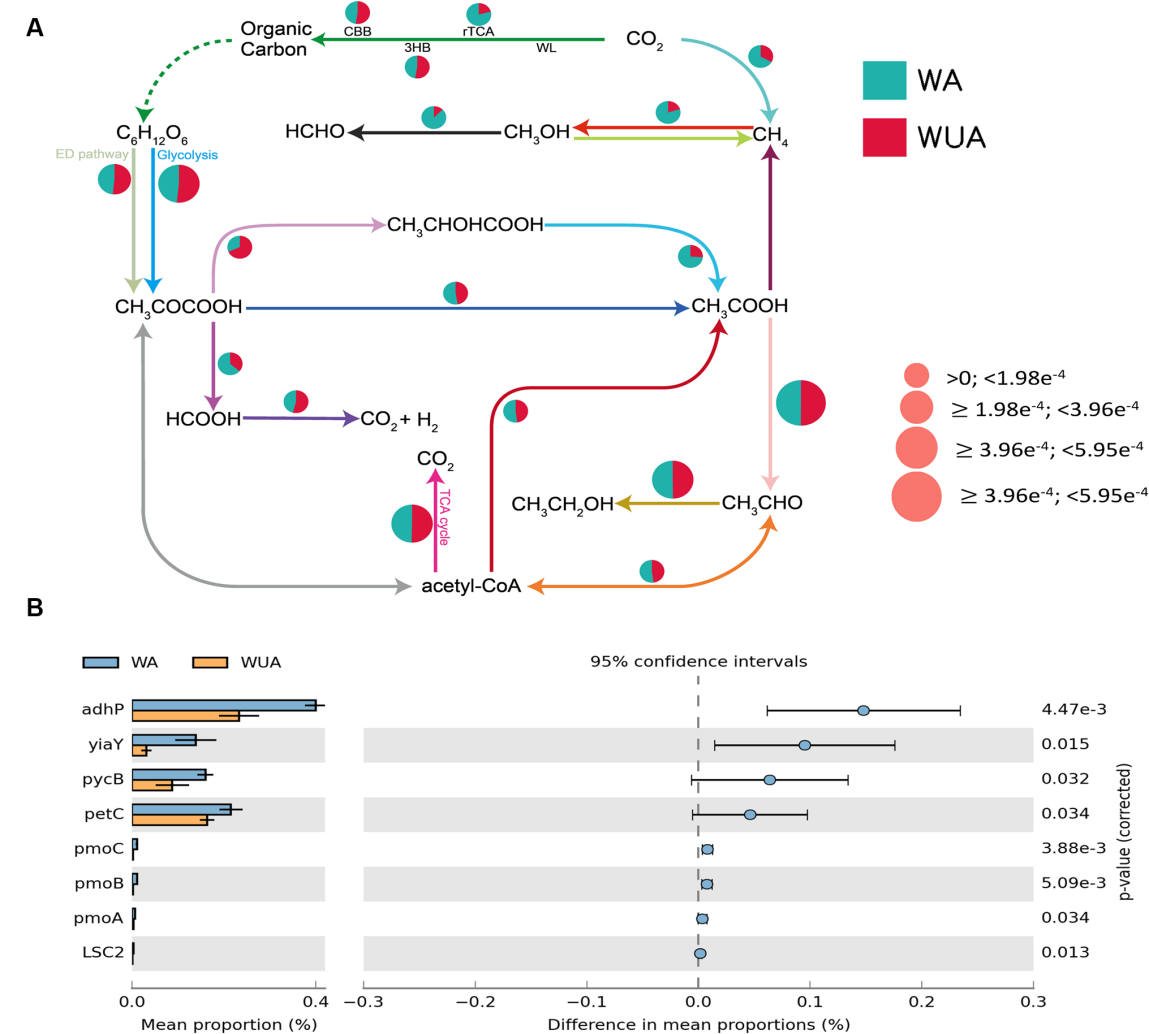
**(A)** Carbon metabolism schematic diagram. **(B)** Key carbon metabolism genes significantly enriched in aquaculture areas.

Through the construction of the carbon metabolism pathway map, it was found that various carbon metabolic pathways had a higher abundance in the aquaculture area, with particular attention to the pathways related to methane metabolism [methano- genesis and anaerobic oxidation of methane (AOM) pathways]. Because studies have shown that eutrophication of seawater regulates the methane-producing microbial community (Meyer-Reil and Köster, 2000). At the same time, several other anaerobic pathways are also significantly more abundant in the aquaculture area compared to the non-aquaculture area, including the reverse tricarboxylic acid cycle (rTCA), lactic acid fermentation (CH3CHOCOOH-CH3COOH), ethanol dehydrogenation (CH3OH-HCHO) and CH3COCOOH-HCOOH (Figure 3A and Supplementary Table S8). Statistical tests of key genes involved in Carbon metabolism were performed by STAMP (v2.0). We observed that all key genes showing significant differences, except for the gene encoding the succinyl-CoA synthetase beta subunit, are associated with anaerobic pathways, including genes encoding alcohol dehydrogenase (*adhP*), alcohol dehydrogenase (*yiaY*), pyruvate carboxylase subunit B (*pycB*), cytochrome b6-f complex iron–sulfur subunit (*petC*) and methane monooxygenase subunit ABC (*pmoABC*) (Figure 3B and Supplementary Table S5).

#### 3.4.2 Nitrogen metabolism

**Figure 4:**
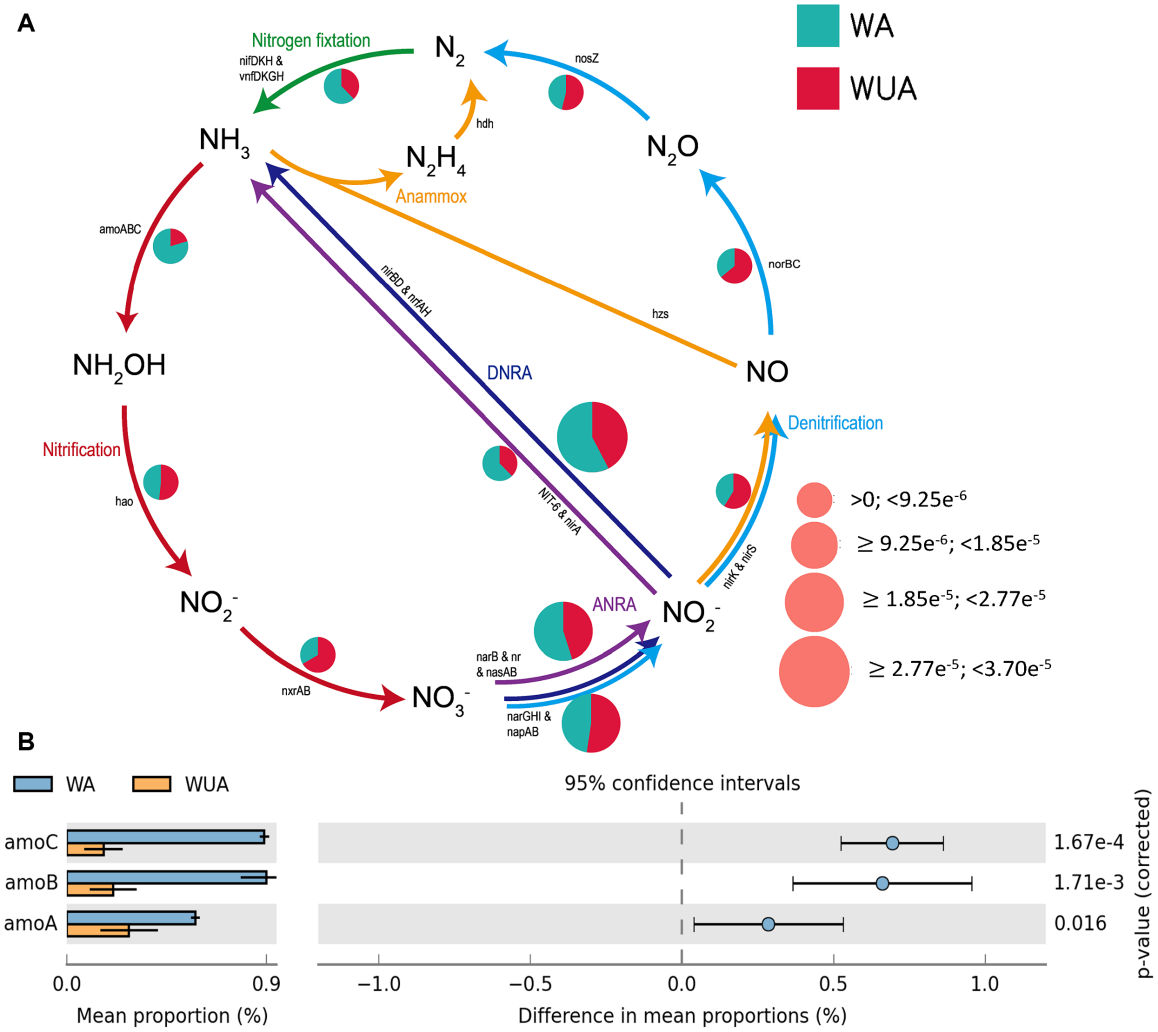
**(A)** Nitrogen metabolism schematic diagram. **(B)** Key Nitrogen metabolism genes significantly enriched in aquaculture areas.

The microbial reactions perceived as predominantly anaerobic, e.g., nitrogen fixation, anaerobic ammonia oxidation (anammox), and dissimilatory nitrate/nitrite reduction to ammonium (DNRA) are all more abundant in aquaculture areas compared to non-aquaculture areas, as shown in the schematic diagram of nitrogen cycling (Figure 4A and Supplementary Table S9). Analysis using STAMP (v2.0) revealed significant differences in key genes related to nitrogen metabolism between aquaculture areas and non-aquaculture areas, with genes encoding ammonia monooxygenase subunit (*amoABC*) significantly enriched in aquaculture areas (Figure 4B and Supplementary Table S6).

#### 3.4.3 Sulfur metabolism

**Figure 5:**
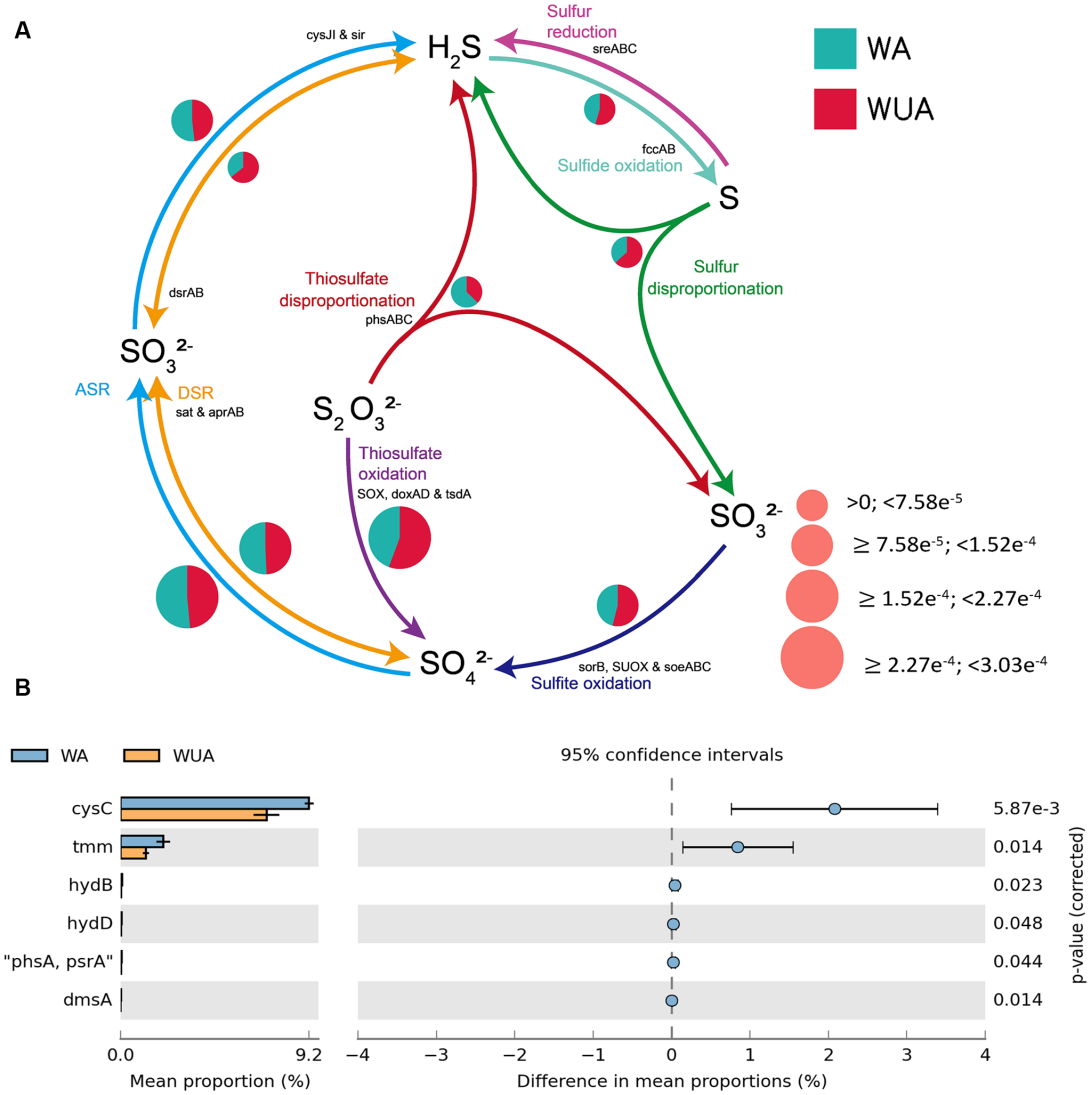
**(A)** Sulfur metabolism schematic diagram. **(B)** Key sulfur metabolism genes significantly enriched in aquaculture areas.

In the schematic diagram of sulfur metabolism element cycling, only the pathway of thiosulfate disproportionation is significantly more abundant in aquaculture areas compared to non-aquaculture areas (Figure 5A and Supplementary Table S10), but this reaction also requires a low-oxygen environment to occur. Analysis of key genes related to sulfur metabolism that show significant differences between aquaculture areas and non-aquaculture areas revealed enrichment of six genes in aquaculture areas, including genes encoding adenylylsulfate kinase (*cysC*), tetrathionate reductase (*ttrC*), sulfhydrogenase (hydD/B), thiosulfate reductase (*phsA*), anaerobic dimethyl sulfoxide reductase (*dmsA*) (Figure 5B and Supplementary Table S7). All of these key genes are also associated with anaerobic sulfur metabolism.

### 3.5 Potential participants in carbon, nitrogen, and sulfur transformation in aquaculture and non-aquaculture areas

**Figure 6:**
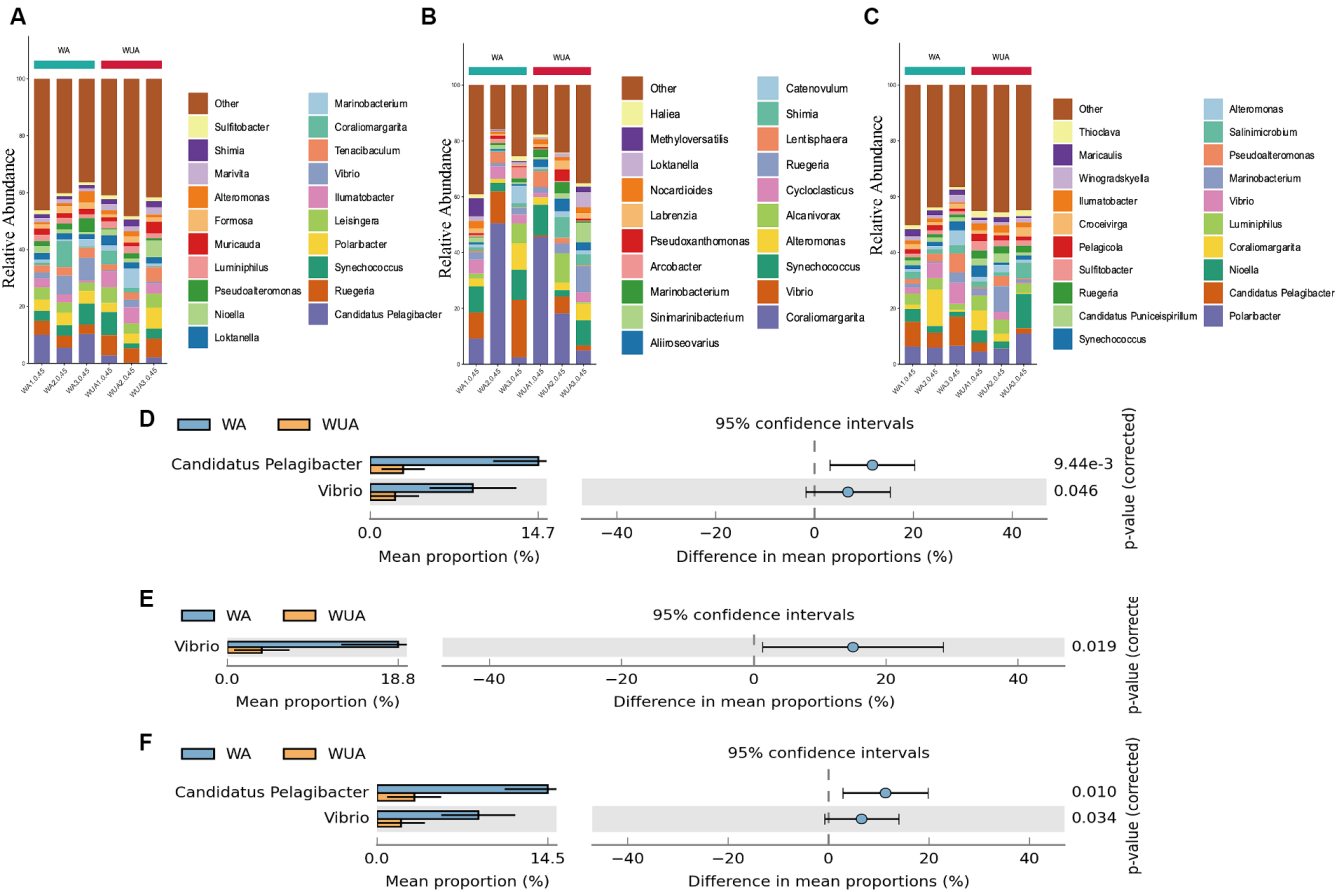
Twenty dominant genera are shown with their relative abundance; the remaining phyla are indicated as “others.” **(A)** Carbon metabolism; **(B)** Nitrogen metabolism; **(C)** Sulfur metabolism. Taxa significantly enriched in aquaculture areas (genus-level annotation). **(D)** Carbon metabolism; **(E)** Nitrogen metabolism; **(F)** Sulfur metabolism.

Key genes related to carbon metabolism, whether in aquaculture or non-aquaculture areas, are taxonomically classified as Proteobacteria (60.07–71.97%) and Bacteroidetes (16.66–32.31%) (Supplementary Figure S2A). At the genus level, significant differences exist in the species participating in carbon metabolism between aquaculture and non-aquaculture areas. *Candidatus Pelagibacter*, and *Vibrio* are significantly more abundant in aquaculture areas, indicating that *Candidatus Pelagibacter*, and *Vibrio*, which are noticeably enriched in aquaculture areas, play a more important role in carbon metabolism (Figures 6A,D).

Key metabolic genes related to nitrogen metabolism are mainly enriched in Proteobacteria (48.29–90.41%) and Verrucomicrobia (1.64–38.97%) in both aquaculture and non-aquaculture areas (Supplementary Figure S2B). Differential analysis at the genus level shows that functional genes related to nitrogen metabolism are more enriched in *Vibrio* in aquaculture areas, indicating that *Vibrio* also plays a more important role in nitrogen metabolism in aquaculture environments (Figures 6B,E).

In sulfur metabolism, key genes are primarily classified as Proteobacteria (61.71–73.87%) and Bacteroidetes (13.62–29.12%), similar to carbon metabolism (Supplementary Figure S2C). At the genus level, *Candidatus Pelagibacter*, and *Vibrio* are significantly more abundant in aquaculture areas, indicating that *Candidatus Pelagibacter*, and *Vibrio*, which are noticeably enriched in aquaculture areas, also play a more important role in sulfur metabolism (Figures 6C,F).

### 3.6 The key environmental factors causing differences in carbon, nitrogen, and sulfur metabolism between aquaculture and non-aquaculture areas

**Figure 7:**
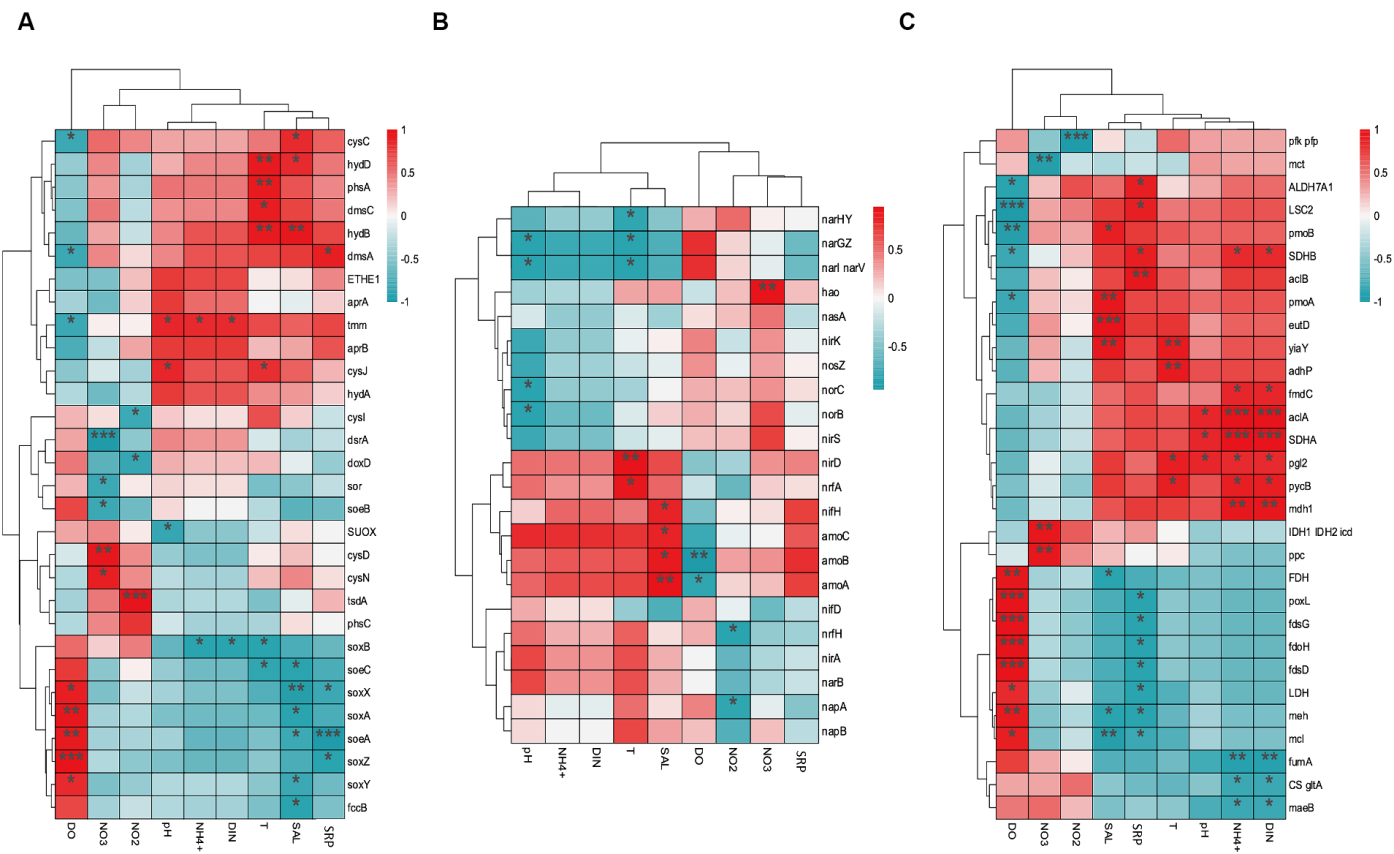
Correlation heat map according to z-scores of the 20 most relevant **(A)** Carbon metabolism **(B)** Nitrogen metabolism **(C)** sulfur metabolism genes with significant correlations among sediment properties. ^*^p < 0.05, ^**^p < 0.01, ^***^p < 0.001.

**Figure 8:**
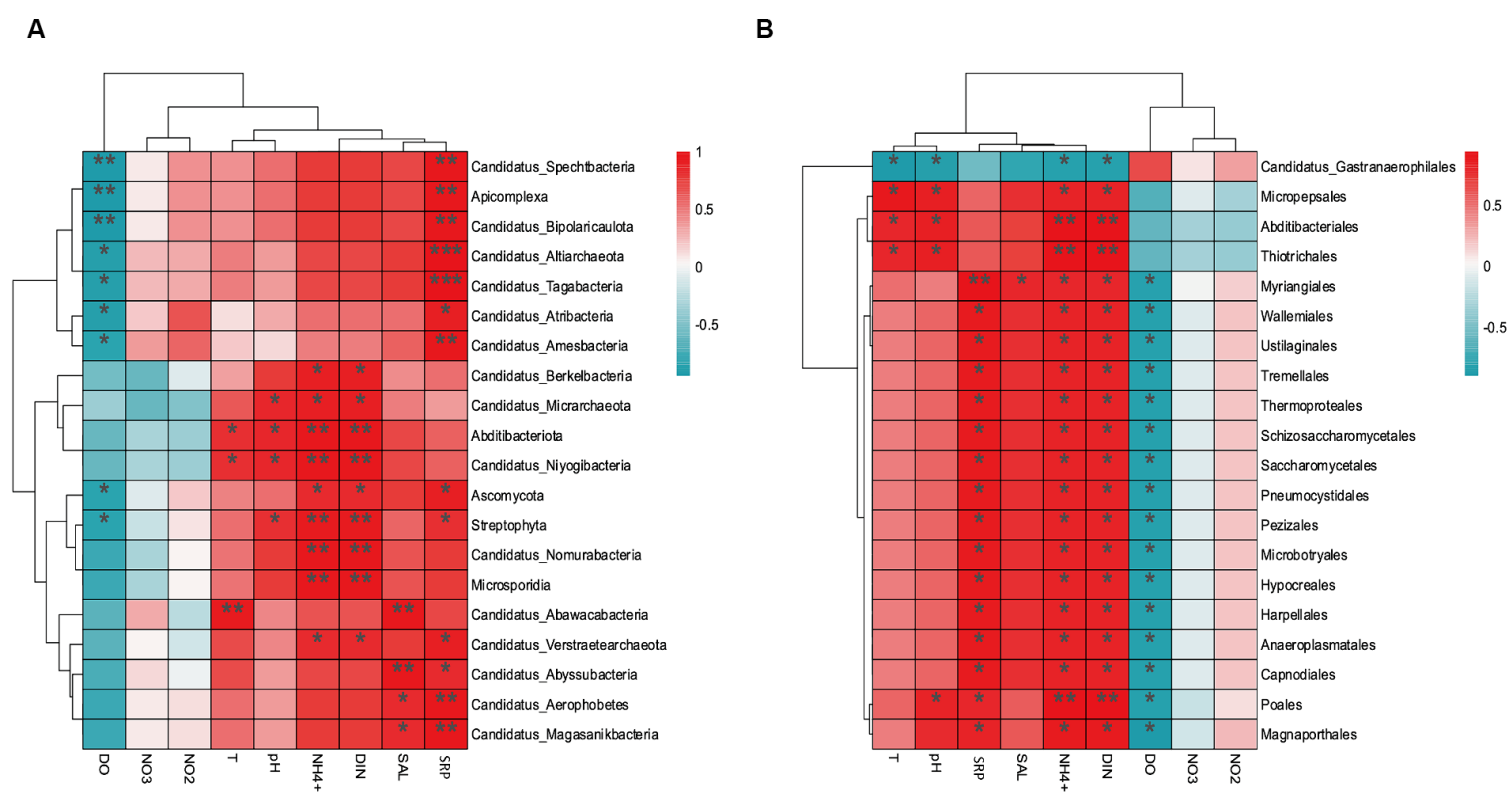
Correlation heat map according to *z*-scores of the 20 most relevant microorganism at the **(A)** phylum level and **(B)** order level. ^*^p < 0.05, ^**^p < 0.01, ^***^p < 0.001.

The environmental factors that likely shaped the structure of carbon bioconversion genes, nitrogen bioconversion genes, sulfur bioconversion genes, and microorganisms in aquaculture and non-aquaculture environments were determined using a correlation heatmap (Figures 7, 8). All environmental factors in carbon metabolism are significantly associated with key genes involved in carbon metabolism, with dissolved oxygen (DO), salinity, soluble reactive phosphorus (SRP), ammonium (NH_4_^+^), and dissolved inorganic nitrogen (DIN) showing stronger and more significant correlations with carbon metabolism-related functional genes. Significantly enriched functional genes related to carbon metabolism in aquaculture areas such as *pmo (A,B)* and *LSC2* are significantly negatively correlated with DO, and several others, even if not significant, also show a negative correlation with DO (Figure 7A). In nitrogen metabolism, only pH, temperature, salinity, DO, nitrate (NO_3_^−^), and nitrite (NO_2_^−^) are significantly associated with nitrogen metabolism, with the *amo (A,B,C)* genes identified in Figure 4B showing significant negative correlation with DO and significant positive correlation with salinity. The dissimilatory nitrate/nitrite reduction to ammonium (DNRA)-related genes *nirD* and *nrfA* identified in Figure 4A also exhibit this correlation, although not significantly (Figure 7B). In sulfur metabolism, all environmental factors are significantly correlated with key genes involved in sulfur metabolism, with dissolved oxygen (DO), nitrate (NO_3_^−^), nitrite (NO_2_^−^), temperature, salinity, and soluble reactive phosphorus (SRP) showing stronger and more significant correlations with sulfur metabolism-related functional genes. Genes such as *cysC*, *tmm*, and *dmsA* identified in Figure 5B are significantly negatively correlated with DO, while others like *hyb(B,D)* and *phsA*, although not significant, also show a negative correlation with DO (Figure 7C). In the correlation analysis between community structure and environmental factors, we found significant associations between dissolved oxygen (DO), temperature, pH, ammonium (NH_4_^+^), dissolved inorganic nitrogen (DIN), soluble reactive phosphorus (SRP), and salinity with microbial communities, with most showing positive correlations with DIN, SRP, NH_4_^+^, and negative correlations with DO (Figure 8).

## 4 Discussion

The microbial diversity in aquatic systems is a vital measure of the environmental quality of aquaculture settings and the health status of aquatic animals (Dantas et al., 2020). It also plays a pivotal role in preserving the ecosystem functions of these environments. In our study, we segmented Lingshui Xincun Port into designated aquaculture and non-aquaculture zones. This division allowed us to conduct a comparative analysis of the variations in microbial diversity within seawater across these areas, encompassing a broad spectrum of microbial taxa. At same time, notable differences were observed between the physicochemical characteristics of the surface water samples collected from aquaculture and non-aquaculture zones (Table 1). Overall, aquaculture zones were more nutrient-rich than non-aquaculture zones. The dissolved inorganic nitrogen (DIN) and soluble reactive phosphorus (SRP) concentration were significantly higher in aquaculture zones than in non-aquaculture zones.

Eutrophication has a significant impact on the cycling of marine nutrients (C, N, P, S, etc.), water quality, biodiversity, and the overall health of coastal marine ecosystems (Ruihua, 1999; Paerl et al., 2003). Bacteria with rapid growth rates, as important components of aquatic environments, can exhibit sensitive and rapid responses to subtle changes in the marine environment (including pollution, physicochemical properties, and biological environment) through their productivity and role in nutrient cycling, thereby serving as indicators (Paerl et al., 2003). The metagenomic sequencing data in this study revealed that Proteobacteria is the predominant phylum across all the samples, aligning with findings from previously published research (Fan et al., 2003; Cardona et al., 2016). In the genus-level annotations, a notable variation in the abundance of species groups was observed between aquaculture and non-aquaculture areas. This disparity may be indicative of the distinct roles these microbial groups play in different environmental contexts (Fan et al., 2017). Analysis using STAMP (v2.0) found that only *Candidatus Pelagibacter* (1.58–28.03%, Supplementary Table S2) was higher in the WA samples than in the WUA in Alphaproteobacteria. Gammaproteobacteria, including *Vibrio* and *Pseudoalteromonas*, was higher in the WA samples than in the WUA samples. The *Candidatus Pelagibacter* are the most abundant heterotrophs in the oceans and are believed to play a major role in mineralizing marine dissolved organic carbon. *Vibrio* and *Pseudoalteromonas* have been proven to be closely associated with eutrophication of seawater. A study on *P. donghaiense* found that as *P. donghaiense* is primarily a photoautotrophic organism with carbon needs mostly fulfilled from uptake of inorganic carbon, the environmental factors regarded to be crucial to its overgrowth during the bloom events are presumably availability of nitrogen and phosphorus, as suggested by previous laboratory observations (Varkitzi et al., 2010; Anderson et al., 2012). Kim et al. (2021) found through their study on the co-proliferation of *Vibrio* spp. and dinoflagellates *Prorocentrum* during a spring algal bloom in the coastal East China Sea that there is a clear correlation between *Vibrio* and algal blooms.

It was found that various carbon metabolic pathways had a higher abundance in the aquaculture area, with particular attention to the pathways related to methane metabolism (methano- genesis and anaerobic oxidation of methane (AOM) pathways). And these pathway-related genes also show a significant negative correlation with dissolved oxygen (DO). Research on the eutrophication of Guanabara Bay in Brazil found that gene libraries based on PCR-DGGE analysis showed that the phylotypes of methanogenic archaea were consistent with the nutrient environment gradient of polluted water bodies. The phylotypes of methanogenic archaea found in anaerobic polluted water bodies and most undamaged water bodies were closely related to known types found in these environments, indicating that water pollution-induced eutrophication factors regulate the methanogenic archaea community (Vieira et al., 2007). Meyer-Reil and Köster (2000) also believe that eutrophic habitats lead to an increase in the population of sulfate-reducing bacteria, while highly anaerobic conditions are conducive to the growth and reproduction of methanogenic archaea, resulting in an increase in the population of methanogenic archaea (Jiang et al., 2009). Therefore, the decrease in redox potential of habitats caused by water eutrophication, the increase in the number of sulfate-reducing bacteria, and the corresponding increase in the number of methanogenic archaea under highly anaerobic conditions are observed.

Nitrogen fixation, anaerobic ammonia oxidation (anammox), and dissimilatory nitrate/nitrite reduction to ammonium (DNRA) are all more abundant in aquaculture areas compared to non-aquaculture areas. In marine surface waters, NO_3_^−^ is typically the most abundant form of nitrogen due to its oxidizing conditions (Patey et al., 2008; Mo et al., 2020). Microbial reactions, such as denitrification, anaerobic ammonia oxidation (anammox), and dissimilatory nitrate/nitrite reduction to ammonium (DNRA), which are predominantly anaerobic, can occur during intermittent hypoxic conditions, such as nocturnal anoxic events during high-biomass algal blooms and periods of extensive biomass decay following the decline of algal blooms (Conley et al., 2011; Turner et al., 2015). These nitrogen transformation reactions mediated by microbes may impact the supply of nitrogen to phytoplankton by influencing nitrogen availability and speciation. Since nitrogen is often considered the limiting nutrient for algal growth during rapid blooms, and previous studies have shown that certain nitrogen species are preferred by *Prorocentrum* spp. over others, investigating these microbially mediated nitrogen transformations could enhance our understanding of bloom dynamics in the East China Sea (Fan et al., 2003; Heil et al., 2005; Varkitzi et al., 2010).

In the schematic diagram of sulfur metabolism element cycling, only the pathway of thiosulfate disproportionation is significantly more abundant in aquaculture areas compared to non-aquaculture areas. Analysis of key genes related to sulfur metabolism that show significant differences between aquaculture areas and non-aquaculture areas revealed enrichment of six genes in aquaculture areas, including genes encoding adenylylsulfate kinase (*cysC*), tetrathionate reductase (*ttrC*), sulfhydrogenase (*hydD/B*), thiosulfate reductase (*phsA*), anaerobic dimethyl sulfoxide reductase (*dmsA*). Sulfate-reducing bacteria are a group of bacteria that use sulfate as the terminal electron acceptor during the oxidation of organic matter, with the end products being H_2_S or S_2_^−^. They are widely distributed in anaerobic sedimentary environments ranging from low to high latitudes (Xuezheng, 2008; Singh et al., 2011). The presence of sulfate-reducing bacteria signifies the occurrence of anaerobic environments, and the population size of these bacteria to some extent reflects the degree of anaerobiosis. Water eutrophication can easily lead to the occurrence of anaerobic environments, and therefore, the population size of sulfate-reducing bacteria can to some extent indicate the level of water nutrient enrichment. Existing studies have shown that in general, the abundance of sulfate-reducing bacteria is negatively correlated with dissolved oxygen content and positively correlated with water nutrient enrichment (Kim et al., 2021).

In summary, the cage aquaculture activities in Lingshui Xincun Port have led to water eutrophication. As the water nutrient levels change, the community structure and function of bacteria also undergo significant changes. The functions of bacteria in the ecosystem also respond to the eutrophication of seawater. The productivity of planktonic bacteria increases with eutrophication, enzyme decomposition potential rises, anaerobic respiration and functional activities are enhanced simultaneously. Eutrophication significantly disrupts the normal functional activity of microorganisms.

In the research on the impact of water eutrophication on bacteria, China’s research still has a certain distance from the world level, and most studies focus on the biomass and productivity of bacteria in freshwater ecosystems and coastal areas. With the rapid development of marine aquaculture, especially cage aquaculture, the trend of water eutrophication caused by marine aquaculture is becoming more serious. This study on the impact of aquaculture-induced water eutrophication on planktonic bacteria provides a new perspective for the management and regulation of eutrophication in aquaculture. And the research provides a basis for environmental management and ecological restoration in eutrophic areas of aquaculture.

## Data availability statement

The datasets presented in this study can be found in online repositories. The names of the repository/repositories and accession number(s) can be found in the article/Supplementary material.

## Ethics statement

All animal-involving experiments of this study were approved by the Animal Care and Use Committee of College of Marine Sciences, South China Agricultural University, and all efforts were made to minimize suffering.

## Author contributions

ZL: Data curation, Formal analysis, Writing – original draft, Writing – review & editing, Investigation, Validation, Visualization. PW: Software, Supervision, Writing – review & editing. JL: Software, Supervision, Writing – review & editing. XL: Software, Writing – review & editing. YZ: Resources, Writing – review & editing. XH: Writing – review & editing. XZ: Resources, Writing – review & editing. WL: Conceptualization, Writing – review & editing. QQ: Conceptualization, Writing – review & editing, Funding acquisition.
