# Supplementary material for: Comparative metagenomic analysis of microbial community compositions and functions in cage aquaculture and its nearby non-aquaculture environments
Source: Front Microbiol. 2024 May 22;15:1398005. doi: 10.3389/fmicb.2024.1398005 (PMC11150647; doi:10.3389/fmicb.2024.1398005)
Supplement: Supplementary file 1 [file Data_Sheet_1.DOCX]

**Table S1**. The key genes of Carbon Metabolism in KEGG database.

| KO | gene | Function |
| --- | --- | --- |
| K00001 | E1.1.1.1, adh | alcohol dehydrogenase |
| K00002 | AKR1A1, adh | alcohol dehydrogenase (NADP+) |
| K00016 | LDH, ldh | L-lactate dehydrogenase |
| K00024 | mdh | malate dehydrogenase |
| K00026 | MDH2 | malate dehydrogenase |
| K00027 | ME2, sfcA, maeA | malate dehydrogenase (oxaloacetate-decarboxylating) |
| K00029 | E1.1.1.40, maeB | malate dehydrogenase (oxaloacetate-decarboxylating)(NADP+) |
| K00030 | IDH3 | isocitrate dehydrogenase (NAD+) |
| K00031 | IDH1, IDH2, icd | isocitrate dehydrogenase |
| K00036 | G6PD, zwf | glucose-6-phosphate 1-dehydrogenase |
| K00114 | exaA | alcohol dehydrogenase (cytochrome c) |
| K00116 | mqo | malate dehydrogenase (quinone) |
| K00121 | frmA, ADH5, adhC | S-(hydroxymethyl)glutathione dehydrogenase / alcohol dehydrogenase |
| K00122 | FDH | formate dehydrogenase |
| K00123 | fdoG, fdhF, fdwA | formate dehydrogenase major subunit |
| K00124 | fdoH, fdsB | formate dehydrogenase iron-sulfur subunit |
| K00126 | fdsD | formate dehydrogenase subunit delta |
| K00127 | fdoI, fdsG | formate dehydrogenase subunit gamma |
| K00128 | ALDH | aldehyde dehydrogenase (NAD+) |
| K00132 | E1.2.1.10 | acetaldehyde dehydrogenase (acetylating) |
| K00138 | aldB | aldehyde dehydrogenase |
| K00156 | poxB | pyruvate dehydrogenase (quinone) |
| K00158 | E1.2.3.3, poxL | pyruvate oxidase |
| K00164 | OGDH, sucA | 2-oxoglutarate dehydrogenase E1 component |
| K00174 | korA, oorA, oforA | 2-oxoglutarate/2-oxoacid ferredoxin oxidoreductase subunit alpha |
| K00175 | korB, oorB, oforB | 2-oxoglutarate/2-oxoacid ferredoxin oxidoreductase subunit beta |
| K00200 | fwdA, fmdA | formylmethanofuran dehydrogenase subunit A |
| K00201 | fwdB, fmdB | formylmethanofuran dehydrogenase subunit B |
| K00202 | fwdC, fmdC | formylmethanofuran dehydrogenase subunit C |
| K00234 | SDHA, SDH1 | succinate dehydrogenase (ubiquinone) flavoprotein subunit |
| K00235 | SDHB, SDH2 | succinate dehydrogenase (ubiquinone) iron-sulfur subunit |
| K00236 | SDHC, SDH3 | succinate dehydrogenase (ubiquinone) cytochrome b560 subunit |
| K00239 | sdhA, frdA | succinate dehydrogenase / fumarate reductase, flavoprotein subunit |
| K00240 | sdhB, frdB | succinate dehydrogenase / fumarate reductase, iron-sulfur subunit |
| K00241 | sdhC, frdC | succinate dehydrogenase / fumarate reductase, cytochrome b subunit |
| K00244 | frdA | fumarate reductase flavoprotein subunit |
| K00245 | frdB | fumarate reductase iron-sulfur subunit |
| K00246 | frdC | fumarate reductase subunit C |
| K00247 | frdD | fumarate reductase subunit D |
| K00382 | DLD, lpd, pdhD | dihydrolipoamide dehydrogenase |
| K00467 | E1.13.12.4 | lactate 2-monooxygenase |
| K00625 | E2.3.1.8, pta | phosphate acetyltransferase |
| K00656 | E2.3.1.54, pflD | formate C-acetyltransferase |
| K00658 | DLST, sucB | 2-oxoglutarate dehydrogenase E2 component (dihydrolipoamide succinyltransferase) |
| K00845 | glk | glucokinase |
| K00846 | KHK | ketohexokinase |
| K00850 | pfkA, PFK | 6-phosphofructokinase 1 |
| K00855 | PRK, prkB | phosphoribulokinase |
| K00873 | PK, pyk | pyruvate kinase |
| K00882 | fruK | 1-phosphofructokinase |
| K00886 | ppgK | polyphosphate glucokinase |
| K00895 | pfp, PFP | diphosphate-dependent phosphofructokinase |
| K01057 | PGLS, pgl, devB | 6-phosphogluconolactonase |
| K01067 | E3.1.2.1, ACH1 | acetyl-CoA hydrolase |
| K01512 | acyP | acylphosphatase |
| K01595 | ppc | phosphoenolpyruvate carboxylase |
| K01596 | E4.1.1.32, pckA, PCK | phosphoenolpyruvate carboxykinase (GTP) |
| K01601 | rbcL | ribulose-bisphosphate carboxylase large chain |
| K01610 | E4.1.1.49, pckA | phosphoenolpyruvate carboxykinase (ATP) |
| K01625 | eda | 2-dehydro-3-deoxyphosphogluconate aldolase / (4S)-4-hydroxy-2-oxoglutarate aldolase |
| K01647 | CS, gltA | citrate synthase |
| K01676 | E4.2.1.2A, fumA, fumB | fumarate hydratase, class I |
| K01677 | E4.2.1.2AA, fumA | fumarate hydratase subunit alpha |
| K01678 | E4.2.1.2AB, fumB | fumarate hydratase subunit beta |
| K01679 | E4.2.1.2B, fumC, FH | fumarate hydratase, class II |
| K01681 | ACO, acnA | aconitate hydratase |
| K01682 | acnB | aconitate hydratase 2 / 2-methylisocitrate dehydratase |
| K01690 | edd | phosphogluconate dehydratase |
| K01899 | LSC1 | succinyl-CoA synthetase alpha subunit |
| K01900 | LSC2 | succinyl-CoA synthetase beta subunit |
| K01902 | sucD | succinyl-CoA synthetase alpha subunit |
| K01903 | sucC | succinyl-CoA synthetase beta subunit |
| K01958 | PC, pyc | pyruvate carboxylase |
| K01959 | pycA | pyruvate carboxylase subunit A |
| K01960 | pycB | pyruvate carboxylase subunit B |
| K02446 | glpX | fructose-1,6-bisphosphatase II |
| K02634 | petA | apocytochrome f |
| K02635 | petB | cytochrome b6 |
| K02636 | petC | cytochrome b6-f complex iron-sulfur subunit |
| K02637 | petD | cytochrome b6-f complex subunit 4 |
| K02640 | petG | cytochrome b6-f complex subunit 5 |
| K02643 | petM | cytochrome b6-f complex subunit 7 |
| K02689 | psaA | photosystem I P700 chlorophyll a apoprotein A1 |
| K02690 | psaB | photosystem I P700 chlorophyll a apoprotein A2 |
| K02691 | psaC | photosystem I subunit VII |
| K02692 | psaD | photosystem I subunit II |
| K02693 | psaE | photosystem I subunit IV |
| K02694 | psaF | photosystem I subunit III |
| K02703 | psbA | photosystem II P680 reaction center D1 protein |
| K02704 | psbB | photosystem II CP47 chlorophyll apoprotein |
| K02705 | psbC | photosystem II CP43 chlorophyll apoprotein |
| K02706 | psbD | photosystem II P680 reaction center D2 protein |
| K02707 | psbE | photosystem II cytochrome b559 subunit alpha |
| K02708 | psbF | photosystem II cytochrome b559 subunit beta |
| K03689 | petN | cytochrome b6-f complex subunit 8 |
| K03841 | FBP, fbp | fructose-1,6-bisphosphatase I |
| K04020 | eutD | phosphotransacetylase |
| K04021 | eutE | aldehyde dehydrogenase |
| K04022 | eutG | alcohol dehydrogenase |
| K04041 | fbp3 | fructose-1,6-bisphosphatase III |
| K04072 | adhE | acetaldehyde dehydrogenase / alcohol dehydrogenase |
| K04073 | mhpF | acetaldehyde dehydrogenase |
| K07404 | pgl | 6-phosphogluconolactonase |
| K08074 | ADPGK | ADP-dependent glucokinase |
| K08691 | mcl | malyl-CoA/(S)-citramalyl-CoA lyase |
| K08928 | pufL | photosynthetic reaction center L subunit |
| K08929 | pufM | photosynthetic reaction center M subunit |
| K09709 | meh | 3-methylfumaryl-CoA hydratase |
| K10944 | pmoA-amoA | methane/ammonia monooxygenase subunit A |
| K10945 | pmoB-amoB | methane/ammonia monooxygenase subunit B |
| K10946 | pmoC-amoC | methane/ammonia monooxygenase subunit C |
| K13788 | pta | phosphate acetyltransferase |
| K13953 | adhP | alcohol dehydrogenase, propanol-preferring |
| K13954 | yiaY | alcohol dehydrogenase |
| K14028 | mdh1, mxaF | methanol dehydrogenase (cytochrome c) subunit 1 |
| K14029 | mdh2, mxaI | methanol dehydrogenase (cytochrome c) subunit 2 |
| K14085 | ALDH7A1 | aldehyde dehydrogenase family 7 member A1 |
| K14468 | mcr | malonyl-CoA reductase / 3-hydroxypropionate dehydrogenase (NADP+) |
| K14469 | K14469 | acrylyl-CoA reductase (NADPH) / 3-hydroxypropionyl-CoA dehydratase / 3-hydroxypropionyl-CoA synthetase |
| K14470 | mct | 2-methylfumaryl-CoA isomerase |
| K15230 | aclA | ATP-citrate lyase alpha-subunit |
| K15231 | aclB | ATP-citrate lyase beta-subunit |
| K16157 | mmoX | methane monooxygenase component A alpha chain |
| K16159 | mmoZ | methane monooxygenase component A gamma chain |
| K16161 | mmoC | methane monooxygenase component C |
| K16370 | pfkB | 6-phosphofructokinase 2 |
| K18118 | aarC, cat1 | succinyl-CoA:acetate CoA-transferase |
| K18366 | bphJ, xylQ, nahO, tesF | acetaldehyde/propanal dehydrogenase |
| K20370 | PEPCK | phosphoenolpyruvate carboxykinase (diphosphate) |
| K21071 | pfk, pfp | ATP-dependent phosphofructokinase / diphosphate-dependent phosphofructokinase |
| K22515 | fdwB | formate dehydrogenase beta subunit |
| K22516 | fdhA | formate dehydrogenase (coenzyme F420) alpha subunit |

**Table S2**. The key genes of Nitrogen metabolism in KEGG database.

| KO | gene | Function |
| --- | --- | --- |
| K00363 | nirD | nitrite reductase (NADH) small subunit |
| K00366 | nirA | ferredoxin-nitrite reductase |
| K00367 | narB | ferredoxin-nitrate reductase |
| K00368 | nirK | nitrite reductase (NO-forming) |
| K00370 | narGZ | nitrate reductase / nitrite oxidoreductaset |
| K00371 | narHY | nitrate reductase / nitrite oxidoreductase |
| K00372 | nasA | assimilatory nitrate reductase catalytic subunit |
| K00374 | narI, narV | nitrate reductase gamma subunit |
| K00376 | nosZ | nitrous-oxide reductase |
| K02305 | norC | nitric oxide reductase subunit C |
| K02567 | napA | nitrate reductase (cytochrome) |
| K02568 | napB | nitrate reductase (cytochrome), electron transfer subunit |
| K02586 | nifD | nitrogenase molybdenum-iron protein alpha chain |
| K02588 | nifH | nitrogenase iron protein NifH |
| K03385 | nrfA | nitrite reductase (cytochrome c-552) |
| K04561 | norB | nitric oxide reductase subunit B |
| K10535 | hao | hydroxylamine dehydrogenase |
| K10944 | amoA | ammonia monooxygenase subunit A |
| K10945 | amoB | ammonia monooxygenase subunit B |
| K10946 | amoC | ammonia monooxygenase subunit C |
| K15864 | nirS | nitrite reductase (NO-forming) / hydroxylamine reductase |
| K15876 | nrfH | cytochrome c nitrite reductase small subunit |

**Table S3**. The key genes of Sulfur metabolism in KEGG database.

| KO | gene | Function |
| --- | --- | --- |
| K00184 | dmsB | dimethyl sulfoxide reductase iron-sulfur subunit |
| K00185 | dmsC | dimethyl sulfoxide reductase membrane subunit |
| K00380 | cysJ | sulfite reductase (NADPH) flavoprotein alpha-component |
| K00381 | cysI | sulfite reductase (NADPH) hemoprotein beta-component |
| K00387 | SUOX | sulfite oxidase |
| K00392 | sir | sulfite reductase (ferredoxin) |
| K00394 | aprA | adenylylsulfate reductase, subunit A |
| K00395 | aprB | adenylylsulfate reductase, subunit B |
| K00860 | cysC | adenylylsulfate kinase |
| K00955 | cysNC | bifunctional enzyme CysN/CysC |
| K00956 | cysN | sulfate adenylyltransferase subunit 1 |
| K00957 | cysD | sulfate adenylyltransferase subunit 2 |
| K00958 | sat | sulfate adenylyltransferase |
| K07306 | dmsA | anaerobic dimethyl sulfoxide reductase subunit A |
| K07307 | dmsB | anaerobic dimethyl sulfoxide reductase subunit B |
| K07308 | dmsC | anaerobic dimethyl sulfoxide reductase subunit C |
| K08352 | phsA, psrA | thiosulfate reductase / polysulfide reductase chain A |
| K08354 | phsC | thiosulfate reductase cytochrome b subunit |
| K11180 | dsrA | dissimilatory sulfite reductase alpha subunit |
| K11181 | dsrB | dissimilatory sulfite reductase beta subunit |
| K16936 | doxA | thiosulfate dehydrogenase (quinone) small subunit |
| K16937 | doxD | thiosulfate dehydrogenase (quinone) large subunit |
| K16952 | sor | sulfur oxygenase/reductase |
| K17222 | soxA | L-cysteine S-thiosulfotransferase |
| K17223 | soxX | L-cysteine S-thiosulfotransferase |
| K17224 | soxB | S-sulfosulfanyl-L-cysteine sulfohydrolase |
| K17226 | soxY | sulfur-oxidizing protein SoxY |
| K17227 | soxZ | sulfur-oxidizing protein SoxZ |
| K17229 | fccB | sulfide dehydrogenase [flavocytochrome c] flavoprotein chain |
| K17230 | fccA | cytochrome subunit of sulfide dehydrogenase |
| K17725 | ETHE1 | sulfur dioxygenase |
| K17993 | hydA | sulfhydrogenase subunit alpha |
| K17994 | hydD | sulfhydrogenase subunit delta |
| K17996 | hydB | sulfhydrogenase subunit beta (sulfur reductase) |
| K18277 | tmm | trimethylamine monooxygenase |
| K19713 | tsdA | thiosulfate dehydrogenase |
| K21307 | soeA | sulfite dehydrogenase (quinone) subunit SoeA |
| K21308 | soeB | sulfite dehydrogenase (quinone) subunit SoeB |
| K21309 | soeC | sulfite dehydrogenase (quinone) subunit SoeC |

**Table S4.** The sequencing quality of Metagenome gene reads for each sample.

|  | WA1 | WA2 | WA3 | WUA1 | WUA2 | WUA3 |
| --- | --- | --- | --- | --- | --- | --- |
| Raw dataM bp(M bp) | 76102868 | 79680432 | 72516962 | 72386378 | 73930870 | 73896630 |
| Clean data(M bp) | 59998044 | 61419830 | 56696470 | 55790016 | 57282166 | 57022126 |
| No. of scafftlgs | 412296 | 415073 | 419242 | 290143 | 322209 | 299748 |
| N50 len.(bp) | 1273 | 1276 | 1209 | 1263 | 1346 | 1344 |
| Predicted ORFs | 732493 | 740977 | 732904 | 529312 | 598055 | 542515 |
| Unigenes | 732281 | 740764 | 732675 | 529131 | 597845 | 542345 |

**Table S5.** Relative abundance of key carbon metabolism genes.

| gene | WA1.0.45 | WA2.0.45 | WA3.0.45 | WUA1.0.45 | WUA2.0.45 | WUA3.0.45 |
| --- | --- | --- | --- | --- | --- | --- |
| aarC, cat1 | 4.32E-08 | 3.01E-07 | 8.57E-08 | 7.10E-08 | 6.73E-07 | 3.77E-08 |
| ACH1 | 6.74E-08 | 2.66E-08 | 3.52E-07 | 1.00E-07 | 1.23E-07 | 5.23E-08 |
| aclA | 2.61E-07 | 6.77E-08 | 5.72E-08 | 1.41E-08 | 1.39E-08 | 7.10E-09 |
| aclB | 1.04E-06 | 4.78E-07 | 5.12E-07 | 2.44E-07 | 3.68E-07 | 2.97E-07 |
| acnB | 7.87E-05 | 8.29E-05 | 9.06E-05 | 8.43E-05 | 9.84E-05 | 7.28E-05 |
| ACO, acnA | 5.55E-05 | 6.05E-05 | 8.26E-05 | 5.70E-05 | 6.46E-05 | 9.00E-05 |
| acyP | 1.73E-05 | 3.20E-05 | 2.03E-05 | 1.19E-05 | 1.48E-05 | 2.20E-05 |
| adh | 1.27E-05 | 1.29E-05 | 2.03E-05 | 1.89E-05 | 3.37E-05 | 1.51E-05 |
| adhE | 4.60E-07 | 1.92E-06 | 3.29E-06 | 4.87E-08 | 1.05E-06 | 1.34E-07 |
| adhP | 1.98E-05 | 2.19E-05 | 2.70E-05 | 1.33E-05 | 1.72E-05 | 1.07E-05 |
| ADPGK | 3.18E-08 | 0 | 3.31E-08 | 8.14E-08 | 0 | 1.31E-07 |
| AKR1A1, adh | 1.57E-06 | 2.90E-06 | 3.20E-06 | 6.14E-07 | 3.84E-06 | 1.10E-06 |
| aldB | 1.31E-05 | 1.52E-05 | 2.53E-05 | 1.18E-05 | 3.09E-05 | 1.03E-05 |
| ALDH | 3.27E-04 | 3.09E-04 | 4.16E-04 | 3.20E-04 | 3.22E-04 | 4.11E-04 |
| ALDH7A1 | 5.95E-07 | 1.04E-07 | 3.60E-07 | 5.21E-08 | 0 | 3.42E-07 |
| bphJ, xylQ, nahO, tesF | 2.05E-05 | 2.06E-05 | 2.23E-05 | 2.20E-05 | 2.26E-05 | 1.80E-05 |
| CS, gltA | 2.70E-04 | 2.87E-04 | 3.03E-04 | 3.17E-04 | 3.01E-04 | 3.18E-04 |
| DLD | 3.37E-04 | 3.68E-04 | 4.19E-04 | 3.69E-04 | 3.58E-04 | 4.29E-04 |
| DLST, sucB | 2.46E-04 | 2.44E-04 | 3.58E-04 | 2.54E-04 | 2.62E-04 | 3.51E-04 |
| E1.13.12.4 | 9.98E-07 | 2.82E-06 | 1.11E-07 | 3.23E-07 | 6.70E-07 | 3.93E-07 |
| E1.2.1.10 | 4.13E-06 | 4.11E-06 | 3.59E-06 | 2.80E-06 | 3.25E-06 | 3.86E-06 |
| E4.1.1.32, pckA, PCK | 8.32E-06 | 1.69E-05 | 5.58E-06 | 1.06E-05 | 1.01E-05 | 5.72E-06 |
| E4.1.1.49, pckA | 1.18E-04 | 1.27E-04 | 1.54E-04 | 1.44E-04 | 1.64E-04 | 1.39E-04 |
| eda | 1.31E-04 | 1.51E-04 | 1.56E-04 | 1.54E-04 | 1.68E-04 | 1.20E-04 |
| edd | 7.08E-05 | 7.53E-05 | 8.34E-05 | 8.92E-05 | 1.08E-04 | 6.96E-05 |
| eutD | 3.22E-07 | 7.86E-07 | 3.26E-07 | 6.70E-08 | 2.14E-07 | 2.19E-07 |
| eutE | 3.17E-06 | 8.37E-06 | 1.65E-06 | 3.02E-06 | 2.00E-06 | 1.00E-06 |
| eutG | 5.55E-08 | 0 | 4.05E-07 | 2.84E-08 | 0 | 0 |
| exaA | 2.22E-05 | 2.31E-05 | 2.52E-05 | 2.27E-05 | 2.76E-05 | 2.25E-05 |
| FBP, fbp | 7.87E-05 | 8.06E-05 | 1.15E-04 | 6.73E-05 | 7.65E-05 | 1.03E-04 |
| fbp3 | 3.68E-08 | 3.99E-07 | 0 | 0 | 9.91E-08 | 0 |
| FDH | 2.37E-06 | 1.72E-06 | 1.07E-06 | 2.99E-06 | 4.57E-06 | 2.77E-06 |
| fdhA | 1.78E-07 | 4.13E-08 | 0 | 0 | 0 | 2.81E-07 |
| fdoG | 8.64E-05 | 8.55E-05 | 9.23E-05 | 9.88E-05 | 1.06E-04 | 8.46E-05 |
| fdoH | 6.65E-05 | 6.99E-05 | 6.35E-05 | 8.48E-05 | 9.08E-05 | 7.16E-05 |
| fdsD | 9.04E-06 | 1.01E-05 | 8.96E-06 | 1.37E-05 | 2.38E-05 | 1.31E-05 |
| fdsG | 8.84E-05 | 9.20E-05 | 8.31E-05 | 1.14E-04 | 1.18E-04 | 9.58E-05 |
| fdwB | 5.19E-05 | 5.18E-05 | 5.90E-05 | 5.69E-05 | 5.22E-05 | 5.27E-05 |
| fmdA | 1.95E-06 | 4.65E-07 | 3.52E-07 | 4.73E-07 | 4.93E-07 | 3.30E-07 |
| fmdB | 8.42E-07 | 7.76E-07 | 2.79E-07 | 4.43E-07 | 4.46E-07 | 2.87E-07 |
| fmdC | 3.12E-07 | 2.79E-08 | 0 | 0 | 0 | 0 |
| frdA | 3.78E-05 | 3.82E-05 | 3.68E-05 | 3.56E-05 | 3.47E-05 | 3.89E-05 |
| frdB | 9.66E-06 | 1.76E-05 | 1.92E-05 | 1.45E-05 | 2.05E-05 | 1.37E-05 |
| frdC | 8.76E-06 | 1.75E-05 | 1.73E-05 | 1.29E-05 | 1.76E-05 | 1.43E-05 |
| frdD | 8.45E-06 | 1.77E-05 | 1.87E-05 | 1.37E-05 | 1.70E-05 | 1.52E-05 |
| frmA, ADH5, adhC | 1.49E-04 | 1.44E-04 | 1.51E-04 | 1.56E-04 | 1.78E-04 | 1.38E-04 |
| fruK | 1.73E-06 | 1.40E-05 | 2.75E-06 | 5.18E-06 | 2.98E-06 | 5.60E-07 |
| fumA | 0 | 0 | 3.43E-08 | 6.74E-08 | 1.66E-07 | 6.79E-08 |
| fumA, fumB | 1.03E-04 | 1.07E-04 | 9.93E-05 | 1.09E-04 | 1.24E-04 | 9.38E-05 |
| fumB | 0 | 3.91E-08 | 2.89E-07 | 1.22E-07 | 0 | 4.09E-08 |
| fumC | 8.40E-05 | 8.77E-05 | 1.56E-04 | 8.61E-05 | 8.40E-05 | 1.33E-04 |
| G6PD | 1.04E-04 | 1.23E-04 | 1.24E-04 | 1.33E-04 | 1.47E-04 | 1.10E-04 |
| glk | 1.99E-04 | 2.00E-04 | 2.02E-04 | 2.26E-04 | 2.32E-04 | 1.84E-04 |
| glpX | 1.14E-05 | 1.47E-05 | 8.01E-06 | 2.33E-05 | 1.59E-05 | 8.49E-06 |
| IDH1, IDH2, icd | 1.84E-04 | 1.82E-04 | 2.20E-04 | 1.76E-04 | 1.93E-04 | 2.16E-04 |
| IDH3 | 2.01E-05 | 1.08E-05 | 2.41E-05 | 1.00E-05 | 1.57E-05 | 1.07E-05 |
| K14469 | 5.20E-07 | 5.21E-07 | 9.28E-07 | 5.23E-07 | 4.94E-07 | 4.44E-07 |
| KHK | 5.56E-06 | 4.42E-06 | 1.06E-06 | 1.20E-05 | 7.67E-07 | 5.54E-06 |
| LDH | 4.32E-06 | 7.96E-06 | 3.92E-06 | 1.24E-05 | 1.04E-05 | 1.20E-05 |
| LSC1 | 1.98E-07 | 3.91E-08 | 0 | 1.02E-07 | 0 | 2.05E-08 |
| LSC2 | 1.53E-07 | 1.16E-07 | 2.28E-07 | 6.95E-08 | 3.39E-08 | 8.76E-08 |
| maeA | 2.09E-05 | 1.73E-05 | 1.93E-05 | 1.54E-05 | 1.94E-05 | 1.26E-05 |
| maeB | 1.72E-04 | 1.69E-04 | 2.10E-04 | 1.98E-04 | 2.31E-04 | 2.24E-04 |
| mcl | 8.14E-05 | 7.96E-05 | 7.21E-05 | 9.66E-05 | 9.18E-05 | 9.04E-05 |
| mcr | 2.32E-06 | 2.24E-06 | 3.72E-06 | 3.02E-06 | 2.99E-06 | 2.29E-06 |
| mct | 6.18E-05 | 5.94E-05 | 5.46E-05 | 6.58E-05 | 5.61E-05 | 5.76E-05 |
| mdh | 2.08E-04 | 2.16E-04 | 2.48E-04 | 2.25E-04 | 2.31E-04 | 2.37E-04 |
| mdh1 | 4.27E-07 | 3.98E-08 | 2.52E-08 | 8.30E-09 | 2.44E-08 | 0 |
| MDH2 | 2.61E-07 | 4.06E-08 | 2.58E-08 | 1.35E-07 | 5.01E-08 | 4.24E-08 |
| mdh2 | 5.79E-07 | 3.36E-08 | 1.07E-07 | 6.97E-08 | 0 | 7.03E-08 |
| meh | 1.05E-04 | 1.05E-04 | 9.63E-05 | 1.22E-04 | 1.10E-04 | 1.09E-04 |
| mhpF | 9.18E-06 | 1.10E-05 | 4.95E-06 | 9.28E-06 | 1.07E-05 | 9.72E-06 |
| mmoC | 0 | 4.82E-07 | 3.82E-08 | 0 | 0 | 1.01E-07 |
| mmoX | 0 | 3.84E-07 | 2.38E-08 | 0 | 1.54E-08 | 7.09E-08 |
| mmoZ | 0 | 4.81E-07 | 3.07E-08 | 0 | 0 | 1.61E-07 |
| mqo | 1.05E-05 | 2.23E-05 | 1.56E-05 | 2.43E-05 | 1.62E-05 | 7.99E-06 |
| oforA | 3.67E-05 | 3.66E-05 | 2.61E-05 | 3.67E-05 | 3.13E-05 | 3.31E-05 |
| oforB | 5.38E-05 | 4.83E-05 | 3.46E-05 | 3.98E-05 | 3.64E-05 | 3.70E-05 |
| PC, pyc | 2.37E-05 | 3.18E-05 | 2.20E-05 | 2.32E-05 | 2.36E-05 | 2.75E-05 |
| PEPCK | 1.41E-06 | 7.82E-06 | 1.45E-06 | 1.88E-06 | 1.18E-06 | 9.37E-07 |
| petA | 1.84E-06 | 2.54E-06 | 5.24E-06 | 5.25E-06 | 1.42E-06 | 1.96E-06 |
| petB | 2.47E-06 | 3.30E-06 | 5.40E-06 | 1.13E-05 | 1.71E-06 | 2.18E-06 |
| petC | 1.24E-05 | 1.00E-05 | 1.45E-05 | 1.07E-05 | 9.46E-06 | 8.68E-06 |
| petD | 1.33E-06 | 2.29E-06 | 3.42E-06 | 3.94E-06 | 1.31E-06 | 1.66E-06 |
| petG | 0 | 1.23E-06 | 0 | 7.27E-07 | 0 | 0 |
| petM | 7.34E-07 | 9.11E-07 | 2.69E-06 | 2.48E-06 | 4.06E-07 | 1.19E-06 |
| petN | 2.97E-07 | 1.96E-07 | 1.03E-07 | 7.11E-07 | 2.00E-07 | 5.12E-07 |
| pfk, pfp | 7.95E-05 | 9.07E-05 | 7.24E-05 | 8.11E-05 | 8.65E-05 | 6.04E-05 |
| pfkA | 7.21E-05 | 5.94E-05 | 1.09E-04 | 6.08E-05 | 7.36E-05 | 1.19E-04 |
| pfkB | 5.75E-06 | 4.58E-06 | 4.21E-06 | 8.00E-06 | 6.91E-06 | 1.25E-05 |
| pflD | 1.01E-06 | 5.63E-06 | 4.21E-06 | 2.04E-06 | 2.52E-06 | 1.71E-06 |
| pfp, PFP | 1.86E-06 | 8.80E-06 | 8.06E-07 | 5.02E-06 | 2.59E-06 | 8.51E-07 |
| pgl1 | 1.13E-04 | 1.09E-04 | 1.33E-04 | 1.37E-04 | 1.48E-04 | 1.23E-04 |
| pgl2 | 1.12E-05 | 3.44E-05 | 1.26E-05 | 9.38E-06 | 8.55E-06 | 5.21E-06 |
| pmoA | 2.78E-07 | 5.28E-07 | 4.02E-07 | 2.04E-07 | 6.69E-08 | 2.66E-07 |
| pmoB | 4.03E-07 | 7.23E-07 | 7.43E-07 | 9.61E-08 | 7.25E-08 | 2.40E-07 |
| pmoC | 4.31E-07 | 7.78E-07 | 6.40E-07 | 1.56E-07 | 3.49E-08 | 1.21E-07 |
| poxB | 4.10E-07 | 3.99E-07 | 3.88E-06 | 1.80E-07 | 5.03E-06 | 1.33E-06 |
| poxL | 2.16E-06 | 2.67E-06 | 8.05E-07 | 4.03E-06 | 5.89E-06 | 2.95E-06 |
| ppc | 5.90E-05 | 7.08E-05 | 1.04E-04 | 7.05E-05 | 8.11E-05 | 8.72E-05 |
| ppgK | 4.07E-05 | 4.02E-05 | 3.44E-05 | 4.64E-05 | 4.08E-05 | 5.26E-05 |
| prkB | 1.56E-05 | 1.66E-05 | 2.99E-05 | 2.53E-05 | 2.75E-05 | 1.52E-05 |
| psaA | 1.49E-06 | 1.92E-06 | 3.50E-06 | 3.58E-06 | 7.19E-07 | 1.15E-06 |
| psaB | 2.03E-06 | 2.62E-06 | 4.80E-06 | 4.24E-06 | 1.18E-06 | 1.43E-06 |
| psaC | 2.36E-06 | 3.27E-06 | 6.15E-06 | 6.17E-06 | 1.63E-06 | 1.98E-06 |
| psaD | 2.22E-06 | 2.26E-06 | 4.61E-06 | 4.97E-06 | 1.06E-06 | 1.59E-06 |
| psaE | 2.03E-06 | 5.00E-06 | 5.58E-06 | 8.43E-06 | 1.99E-06 | 1.93E-06 |
| psaF | 1.86E-06 | 3.23E-06 | 6.48E-06 | 4.86E-06 | 1.60E-06 | 1.27E-06 |
| psbA | 2.61E-05 | 1.91E-05 | 2.07E-05 | 2.53E-05 | 1.45E-05 | 8.26E-06 |
| psbB | 2.05E-06 | 2.77E-06 | 5.78E-06 | 5.64E-06 | 9.27E-07 | 1.45E-06 |
| psbC | 1.94E-06 | 2.91E-06 | 4.80E-06 | 4.47E-06 | 1.01E-06 | 2.12E-06 |
| psbD | 1.48E-05 | 1.31E-05 | 1.31E-05 | 1.61E-05 | 7.62E-06 | 4.89E-06 |
| psbE | 2.26E-06 | 3.27E-06 | 5.13E-06 | 5.57E-06 | 1.35E-06 | 2.10E-06 |
| psbF | 2.66E-06 | 3.09E-06 | 6.65E-06 | 5.24E-06 | 8.37E-07 | 2.12E-06 |
| pta1 | 9.14E-05 | 9.61E-05 | 9.37E-05 | 1.02E-04 | 9.86E-05 | 8.18E-05 |
| pta2 | 7.17E-06 | 1.95E-05 | 1.19E-05 | 8.95E-06 | 1.01E-05 | 1.07E-05 |
| pufL | 6.32E-05 | 5.82E-05 | 5.93E-05 | 7.12E-05 | 6.95E-05 | 5.60E-05 |
| pufM | 7.11E-05 | 6.35E-05 | 6.68E-05 | 7.78E-05 | 7.67E-05 | 6.65E-05 |
| pycA | 9.11E-06 | 8.47E-06 | 9.11E-06 | 5.70E-06 | 9.01E-06 | 3.38E-06 |
| pycB | 9.26E-06 | 9.39E-06 | 8.44E-06 | 4.87E-06 | 7.98E-06 | 2.68E-06 |
| pyk | 1.99E-04 | 2.16E-04 | 2.47E-04 | 2.29E-04 | 2.44E-04 | 2.41E-04 |
| rbcL | 6.23E-05 | 5.13E-05 | 3.70E-05 | 7.04E-05 | 5.79E-05 | 5.71E-05 |
| SDHA | 8.06E-07 | 3.43E-07 | 2.16E-07 | 6.01E-08 | 3.86E-08 | 2.12E-08 |
| sdhA, frdA | 1.67E-04 | 1.70E-04 | 2.20E-04 | 1.81E-04 | 1.77E-04 | 1.92E-04 |
| SDHB | 9.36E-06 | 3.80E-06 | 2.15E-06 | 2.16E-07 | 1.29E-07 | 9.43E-07 |
| sdhB, frdB | 2.03E-04 | 2.14E-04 | 2.47E-04 | 2.10E-04 | 2.10E-04 | 2.29E-04 |
| SDHC | 3.51E-07 | 6.95E-08 | 1.07E-07 | 2.62E-08 | 0 | 2.62E-07 |
| sdhC, frdC | 1.90E-04 | 2.12E-04 | 2.48E-04 | 2.12E-04 | 2.20E-04 | 2.26E-04 |
| sucA | 9.94E-05 | 1.08E-04 | 1.21E-04 | 9.53E-05 | 1.25E-04 | 1.24E-04 |
| sucC | 2.25E-04 | 0.00022738 | 2.76E-04 | 2.38E-04 | 2.28E-04 | 2.47E-04 |
| sucD | 2.05E-04 | 2.20E-04 | 2.44E-04 | 2.20E-04 | 2.23E-04 | 2.31E-04 |
| yiaY | 4.23E-06 | 1.05E-05 | 9.28E-06 | 1.30E-06 | 2.84E-06 | 1.43E-06 |

**Table S6.** Relative abundance of key nitrogen metabolism genes.

| gene | WA1.0.45 | WA2.0.45 | WA3.0.45 | WUA1.0.45 | WUA2.0.45 | WUA3.0.45 |
| --- | --- | --- | --- | --- | --- | --- |
| nirD | 1.65E-05 | 1.87E-05 | 2.47E-05 | 1.47E-05 | 1.69E-05 | 1.46E-05 |
| nirA | 2.85E-06 | 9.35E-06 | 4.65E-06 | 5.78E-06 | 2.96E-06 | 1.45E-06 |
| narB | 1.68E-06 | 9.20E-06 | 3.51E-06 | 6.70E-06 | 2.48E-06 | 1.44E-06 |
| nirK | 1.63E-06 | 1.94E-06 | 1.12E-06 | 3.68E-07 | 3.04E-06 | 2.24E-06 |
| narGZ | 2.39E-06 | 1.69E-06 | 1.56E-06 | 2.62E-06 | 3.28E-06 | 7.89E-06 |
| narHY | 5.07E-06 | 2.75E-06 | 3.39E-06 | 3.66E-06 | 4.85E-06 | 1.14E-05 |
| nasA | 5.86E-06 | 5.78E-06 | 1.38E-05 | 7.94E-06 | 1.16E-05 | 6.21E-06 |
| narI, narV | 3.96E-06 | 3.00E-06 | 2.13E-06 | 4.11E-06 | 4.92E-06 | 1.05E-05 |
| nosZ | 1.05E-06 | 7.38E-07 | 6.24E-07 | 3.95E-07 | 1.28E-06 | 1.15E-06 |
| norC | 4.37E-07 | 3.70E-07 | 3.54E-07 | 3.53E-07 | 8.35E-07 | 1.53E-06 |
| napA | 1.74E-06 | 6.90E-06 | 2.39E-06 | 2.54E-06 | 2.70E-06 | 1.82E-06 |
| napB | 2.96E-06 | 1.10E-05 | 5.62E-06 | 4.51E-06 | 5.66E-06 | 4.38E-06 |
| nifD | 2.49E-07 | 4.90E-08 | 6.89E-08 | 2.54E-07 | 1.51E-07 | 6.82E-08 |
| nifH | 3.60E-07 | 5.33E-07 | 1.15E-07 | 5.93E-08 | 8.65E-08 | 2.14E-07 |
| nrfA | 2.22E-06 | 7.98E-06 | 4.40E-06 | 2.79E-06 | 2.95E-06 | 6.56E-07 |
| norB | 4.96E-07 | 1.56E-06 | 5.51E-07 | 4.28E-07 | 1.77E-06 | 2.03E-06 |
| hao | 1.13E-07 | 1.38E-07 | 3.66E-07 | 1.07E-07 | 2.86E-07 | 2.64E-07 |
| amoA | 2.78E-07 | 5.28E-07 | 4.02E-07 | 2.04E-07 | 6.69E-08 | 2.66E-07 |
| amoB | 4.03E-07 | 7.23E-07 | 7.43E-07 | 9.61E-08 | 7.25E-08 | 2.40E-07 |
| amoC | 4.31E-07 | 7.78E-07 | 6.40E-07 | 1.56E-07 | 3.49E-08 | 1.21E-07 |
| nirS | 3.72E-07 | 3.58E-07 | 6.92E-07 | 1.83E-07 | 1.59E-06 | 1.29E-06 |
| nrfH | 1.06E-06 | 6.94E-06 | 1.18E-06 | 3.64E-06 | 1.91E-06 | 4.32E-07 |

**Table S7.** Relative abundance of key sulfur metabolism genes.

| gene | WA1.0.45 | WA2.0.45 | WA3.0.45 | WUA1.0.45 | WUA2.0.45 | WUA3.0.45 |
| --- | --- | --- | --- | --- | --- | --- |
| tsdA | 3.71E-05 | 2.54E-05 | 6.21E-05 | 3.34E-05 | 2.90E-05 | 8.93E-05 |
| tmm | 2.96E-05 | 2.46E-05 | 3.87E-05 | 2.29E-05 | 2.03E-05 | 1.67E-05 |
| SUOX | 1.18E-05 | 1.28E-05 | 9.99E-06 | 8.32E-06 | 1.29E-05 | 1.83E-05 |
| soxZ | 8.89E-05 | 8.94E-05 | 8.12E-05 | 1.22E-04 | 1.23E-04 | 9.17E-05 |
| soxY | 9.97E-05 | 9.58E-05 | 8.76E-05 | 1.29E-04 | 1.32E-04 | 9.90E-05 |
| soxX | 9.49E-05 | 9.24E-05 | 8.17E-05 | 1.27E-04 | 1.25E-04 | 9.81E-05 |
| soxB | 6.99E-05 | 6.65E-05 | 8.64E-05 | 9.83E-05 | 9.67E-05 | 9.99E-05 |
| soxA | 8.88E-05 | 8.66E-05 | 8.13E-05 | 1.17E-04 | 1.18E-04 | 9.16E-05 |
| sor | 6.39E-07 | 4.84E-07 | 9.01E-08 | 1.40E-06 | 1.32E-07 | 5.60E-07 |
| soeC | 7.11E-05 | 6.92E-05 | 6.75E-05 | 9.23E-05 | 7.49E-05 | 7.58E-05 |
| soeB | 7.50E-05 | 7.29E-05 | 6.77E-05 | 9.45E-05 | 8.12E-05 | 7.26E-05 |
| soeA | 2.71E-05 | 2.85E-05 | 2.71E-05 | 4.06E-05 | 3.63E-05 | 3.08E-05 |
| sir | 6.73E-06 | 1.07E-05 | 1.10E-05 | 1.23E-05 | 1.36E-05 | 6.79E-06 |
| sat | 4.61E-05 | 3.27E-05 | 3.22E-05 | 4.53E-05 | 3.74E-05 | 4.10E-05 |
| phsC | 9.07E-08 | 9.71E-08 | 2.84E-07 | 2.45E-07 | 5.10E-08 | 2.85E-07 |
| phsA | 2.69E-07 | 5.27E-07 | 7.81E-07 | 1.68E-07 | 3.20E-07 | 1.41E-07 |
| hydD | 5.20E-07 | 1.08E-06 | 8.34E-07 | 3.70E-07 | 6.03E-07 | 4.18E-07 |
| hydB | 5.11E-07 | 1.26E-06 | 8.68E-07 | 1.52E-07 | 3.52E-07 | 2.58E-07 |
| hydA | 1.57E-06 | 2.25E-06 | 1.16E-06 | 1.58E-06 | 1.03E-06 | 1.05E-06 |
| fccB | 6.81E-05 | 5.99E-05 | 6.32E-05 | 7.42E-05 | 8.31E-05 | 6.44E-05 |
| fccA | 1.89E-06 | 5.89E-07 | 3.40E-06 | 1.54E-06 | 1.12E-05 | 2.28E-06 |
| ETHE1 | 2.88E-06 | 1.51E-06 | 2.01E-06 | 2.55E-06 | 1.18E-06 | 1.22E-06 |
| dsrB | 4.12E-06 | 3.18E-06 | 1.31E-06 | 9.84E-06 | 1.24E-06 | 4.70E-06 |
| dsrA | 7.21E-06 | 6.75E-06 | 1.74E-06 | 1.64E-05 | 5.80E-06 | 5.62E-06 |
| doxD | 4.15E-06 | 1.17E-05 | 1.57E-06 | 7.78E-06 | 6.34E-06 | 2.98E-06 |
| doxA | 4.56E-07 | 0 | 2.21E-08 | 5.43E-08 | 0 | 0 |
| dmsC | 1.26E-07 | 3.32E-07 | 4.81E-07 | 0 | 2.83E-07 | 0 |
| dmsB | 5.52E-05 | 6.24E-05 | 5.82E-05 | 5.31E-05 | 4.39E-05 | 8.43E-05 |
| dmsA | 1.21E-07 | 9.57E-08 | 1.35E-07 | 0 | 8.17E-08 | 2.50E-08 |
| dms | 6.32E-05 | 7.04E-05 | 7.79E-05 | 6.02E-05 | 5.19E-05 | 9.39E-05 |
| cysN | 3.99E-05 | 6.67E-05 | 1.09E-04 | 5.63E-05 | 6.03E-05 | 8.23E-05 |
| cysJ | 1.66E-05 | 3.10E-05 | 2.39E-05 | 2.05E-05 | 1.46E-05 | 8.32E-06 |
| cysI | 6.51E-05 | 7.90E-05 | 7.75E-05 | 7.00E-05 | 8.48E-05 | 6.14E-05 |
| cysD | 1.42E-04 | 1.48E-04 | 1.86E-04 | 1.44E-04 | 1.53E-04 | 1.70E-04 |
| cysC | 1.28E-04 | 1.38E-04 | 1.43E-04 | 1.11E-04 | 1.05E-04 | 1.29E-04 |
| cys | 5.38E-05 | 4.39E-05 | 4.12E-05 | 4.59E-05 | 5.66E-05 | 5.15E-05 |
| aprB | 1.59E-05 | 1.07E-05 | 1.39E-05 | 1.36E-05 | 1.14E-06 | 6.01E-06 |
| aprA | 1.14E-05 | 7.30E-06 | 9.89E-06 | 1.25E-05 | 9.41E-07 | 5.42E-06 |

**Table S8.** Carbon metabolism-related pathways and their corresponding KO numbers.

| Pathway | Genes |
| --- | --- |
| Photosystem II (psbABCDEF) | K02703,K02704,K02705,K02706,K02707,K02708 |
| Photosystem I (psaABCDEF) | K02689,K02690,K02691,K02692,K02693,K02694 |
| Cytochrome b6/f complex (petABCDGLMN) | K02635,K02637,K02634,K02636,K02642,K02643,K03689,K02640 |
| Anoxygenic photosystem II (pufML) | K08928,K08929 |
| RuBisCo | K01601 |
| CBB cycle (prkB) | K00855 |
| rTCA cycle (aclAB, ccsAB, ccl) | K15230,K15231,K15232,K15233,K15234 |
| 3-Hydroxypropionate Bicycle | K14468,K15052,K08691,K14469,K14470,K09709 |
| Glycolysis (glk, pfk, pyk) | K00845,K00844,K00918,K00886,K08074,K00846,K00882,K00918,K00895,K21071,K00850,K16370,K00873 |
| Entner-Doudoroff pathway, glucose-6P -> glyceraldehyde-3P + pyruvate | K00036,K01057,K07404,K01690,K01625 |
| Gluconeogenesis (fbp, pck) | K03841,K02446,K04041,K01622,K00895,K21071,K01610,K01596 |
| TCA cycle | K01647,K05942,K01681,K01682,K00031,K00030,K00174,K00175,K00164,K00658,K00382,K01902,K01903,K01899,K01900,K18118,K00234,K00235,K00236,K00237,K00239,K00240,K00241,K00244,K00245,K00246,K01676,K01679,K01677,K01678,K00026,K00025,K00024,K00116 |
| Methanogenesis, CO2 -> methane | K00200,K00201,K00202,K00203,K00205,K11260,K00204 |
| Methane oxidation, methane -> methanol (mmoBCDXYZ, amoABC) | K16157,K16158,K16159,K16160,K16161,K16162,K10944,K10945,K10946 |
| Methane oxidation, methanol -> formaldehyde (mxaFI, xoxF) | K14028,K14029,K17066 |
| Fermentation to lactate, pyruvate -> lactate (LDH) | K00016 |
| Fermentation to formate, pyruvate -> formate (pf1D) | K00656 |
| Fermentation to formate -> CO2 & H2 (fdh) | K00122,K00123,K00124,K00126,K00127,K22515,K22516,K00125 |
| Fermentation to acetate, pyruvate -> acetate (poxB, poxL, acyP) | K00156,K00158,K01512 |
| Fermentation to acetate, acetyl-CoA -> acetate (ach1, eutD, pta, acyP) | K01067,K04020,K13788,K00625,K01512 |
| Fermentation to acetate, lactate -> acetate (EC:1.13.12.4) | K00467 |
| Fermentation to ethanol, acetate to acetylaldehyde (ald) | K00128,K14085,K00149,K00129,K00138 |
| Fermentation to ethanol, acetyl-CoA to acetylaldehyde (reversible) | K00132,K04072,K04073,K18366,K04021 |
| Fermentation to ethanol, acetylaldehyde to ethanol (adh, mdh) | K13951,K13980,K13952,K13953,K13954,K00001,K00121,K04072,K18857,K14028,K14029,K00114,K00002,K04022 |
| Fermentation to succinate (phosphoenolpyruvate to succinate via oxaloacetate, malate & fumarate) | K01595,K01596,K20370,K01610,K00024,K00025,K00026,K00051,K00116,K01676,K01679,K01677,K01678,K00244,K00245,K00246,K00247 |
| Anaplerotic genes (pyruvate -> oxaloacetate) | K00027,K00028,K00029,K01958,K01959,K01960,K01595,K01610,K01596,K20370 |

**Table S9.** Nitrogen metabolism-related pathways and their corresponding KO numbers.

| Pathway | Genes |
| --- | --- |
| Dissimilatory nitrate reduction, nitrate -> nitrite (narGHI or napAB) | K00370,K00371,K00374,K02567,K02568 |
| Dissimilatory nitrate reduction, nitrite -> ammonia (nirBD or nrfAH) | K00362,K00363,K03385,K15876 |
| Assimilatory nitrate reduction, nitrate -> nitrite (narB or NR or nasAB) | K00367,K10534,K00372,K00360 |
| Assimilatory nitrate reduction, nitrite -> ammonia (NIT-6 or nirA) | K17877,K00366 |
| Denitrification, nitrite -> nitric oxide (nirK or nirS) | K00368,K15864 |
| Denitrification, nitric oxide -> nitrous oxide (norBC) | K04561,K02305 |
| Denitrification, nitrous oxide -> nitrogen (nosZ) | K00376 |
| Nitrogen fixation, nitrogen -> ammonia (nifKDH) | K02586,K02588,K02591,K22896,K22897,K22898,K22899 |
| Nitrification, ammonia -> hydroxylamine (amoABC) | K10944,K10945,K10946 |
| Nitrification, hydroxylamine -> nitrite (hao) | K10535 |
| Nitrification, nitrite -> nitrate (nxrAB) | K00370,K00371 |

**Table S10.** Sulfur metabolism-related pathways and their corresponding KO numbers.

| Pathway | Genes |
| --- | --- |
| Assimilatory sulfate reduction, sulfate -> sulfite | K13811,K00958,K00860,K00955,K00957,K00956,K00957,K00860,K13911 |
| Assimilatory sulfate reduction, sulfite -> sulfide (cysJI or sir) | K00380,K00381,K00392 |
| Dissimilatory sulfate reduction, sulfate -> sulfite (reversible) (sat and aprAB) | K00956,K00957,K00958,K00394,K00395 |
| Dissimilatory sulfate reduction, sulfite -> sulfide (reversible) (dsrAB) | K11180,K11181 |
| Thiosulfate oxidation by SOX complex, thiosulfate -> sulfate | K17222,K17223,K17224,K17226,K17227 |
| Alternative thiosulfate oxidation (doxAD) | K16936,K16937 |
| Alternative thiosulfate oxidation (tsdA) | K19713 |
| Thiosulfate oxidation (SOX, doxAD and tsdA) | K17222,K17223,K17224,K17226,K17227,K16936,K16937,K19713 |
| Thiosulfate disproportionation, thiosulfate -> sulfide & sulfite (phsABC) | K08352,K08353,K08354 |
| Sulfhydrogenase, (sulfide)n -> (sulfide)n-1 | K17993,K17994,K17995,K17996 |
| Sulfur disproportionation, sulfur -> sulfide & sulfite | K16952 |
| Sulfur dioxygenase | K17725 |
| Sulfite oxidation, sulfite -> sulfate (sorB, SUOX, soeABC) | K05301,K00387,K21307,K21308,K21309 |
| Sulfide oxidation, sulfide -> sulfur (fccAB) | K17229,K17230 |
| DMS oxidation, DMS -> DMSO (ddhABC or tmm) | K16964,K16965,K16966,K18277 |
| DMSO reduction, DMSO -> DMS (dms or dorA) | K00184,K00185,K07306,K07307,K07308,DorA |

**Table S11.** Relative abundance of key phosphorus metabolism genes.

| gene | WA1.0.45 | WA2.0.45 | WA3.0.45 | WUA1.0.45 | WUA2.0.45 | WUA3.0.45 |
| --- | --- | --- | --- | --- | --- | --- |
| phoB | 4.28E-05 | 5.70E-05 | 5.88E-05 | 3.84E-05 | 5.07E-05 | 3.43E-05 |
| phoD | 1.32E-04 | 1.45E-04 | 1.31E-04 | 8.17E-05 | 1.04E-04 | 1.20E-04 |
| pstB | 1.57E-04 | 1.84E-04 | 1.71E-04 | 1.89E-04 | 1.88E-04 | 1.31E-04 |
| pstC | 1.40E-04 | 1.65E-04 | 1.52E-04 | 1.85E-04 | 1.84E-04 | 1.24E-04 |
| pstA | 1.43E-04 | 1.64E-04 | 1.55E-04 | 1.68E-04 | 1.74E-04 | 1.18E-04 |
| phnC | 5.75E-05 | 6.58E-05 | 6.78E-05 | 6.62E-05 | 5.95E-05 | 4.96E-05 |
| phnE | 9.72E-05 | 1.09E-04 | 1.06E-04 | 1.08E-04 | 1.01E-04 | 7.99E-05 |
| phnF | 1.95E-05 | 1.94E-05 | 2.33E-05 | 2.25E-05 | 2.14E-05 | 1.84E-05 |
| phnD | 8.32E-05 | 8.46E-05 | 9.57E-05 | 8.57E-05 | 9.35E-05 | 7.70E-05 |
| ugpB | 8.70E-05 | 8.34E-05 | 8.62E-05 | 9.39E-05 | 9.27E-05 | 7.66E-05 |
| ugpA | 9.79E-05 | 9.35E-05 | 9.27E-05 | 1.05E-04 | 1.02E-04 | 8.30E-05 |
| ugpE | 7.94E-05 | 7.25E-05 | 8.06E-05 | 7.76E-05 | 7.94E-05 | 6.97E-05 |
| ugpC | 1.40E-04 | 1.36E-04 | 1.44E-04 | 1.48E-04 | 1.39E-04 | 1.25E-04 |

**Table S12.** Relative abundance of virulence genes genes.

| Cluster | WA1.0.45 | WA2.0.45 | WA3.0.45 | WUA1.0.45 | WUA2.0.45 | WUA3.0.45 |
| --- | --- | --- | --- | --- | --- | --- |
| Defensive_virulence_factors | 0.0025123263 | 0.0031633776 | 0.0027687919 | 0.0027142015 | 0.0027537692 | 0.0025944857 |
| Nonspecific_virulence_factor | 0.0004583387 | 0.0004731299 | 0.0005388017 | 0.0004941873 | 0.0005138348 | 0.0004891338 |
| Offensive_virulence_factors | 0.0025536936 | 0.0029871764 | 0.0030115743 | 0.0024521477 | 0.002828129 | 0.0024247578 |
| Regulation_of_virulence-associated_genes | 0.0007527429 | 0.0007475088 | 0.0008320545 | 0.0007834911 | 0.0008327596 | 0.0007633845 |

|  |  | dissolved oxygen | pH | Salinity (‰) | NO_3_^-^-N (mg N /L) | NO_2_^-^-N (mg N /L) | NH_4_^+^-N (mg N /L) | Dissolved inorganic nitrogen (mg N /L) | Dissolved inorganic phosphorus(mg N /L) | Conductivity (µS/cm) | Oxidation-Reduction potential (mV) |
| --- | --- | --- | --- | --- | --- | --- | --- | --- | --- | --- | --- |
| WA | mean | 6.55333333 | 7.8 | 33.5866667 | 0.08 | 0.01166667 | 0.08066667 | 0.77966667 | 0.08656667 | 46122.3333 | 45.7333333 |
|  | SD | 0.40941693 | 0 | 0.01699673 | 0.01920069 | 0.00555778 | 0.00817856 | 0.08106016 | 0.00736357 | 175.623334 | 0.37712362 |
|  | range | 6.22-7.13 | 7.8 | 33.57-33.61 | 0.064-0.107 | 0.004-0.017 | 0.073-0.092 | 0.7037-0.892 | 0.0779-0.0959 | 45963-46367 | 45.2-46 |
| WUA | mean | 3.48137513 | 5.2 | 16.8018317 | 0.04960035 | 0.00861223 | 0.04442262 | 0.43036342 | 0.04696512 | 23148.9783 | 23.0552285 |
|  | SD | 3.0719582 | 3.67695526 | 16.784835 | 0.03039966 | 0.00305445 | 0.03624406 | 0.34930326 | 0.03960155 | 22973.355 | 22.6781048 |
|  | range | 7.37-7.59 | 7.5-7.8 | 32.73-32.91 | 0.059-0.093 | 0.004-0.019 | 0.055-0.069 | 0.5252-0.664 | 0.0317-0.0558 | 47063-47246 | 40.5-45 |

**Table S13.** physicochemical characteristics of Aquaculture area and non-aquaculture area
