# Supplementary material for: Comparative metagenomic analysis of microbial community compositions and functions in cage aquaculture and its nearby non-aquaculture environments
Source: Front Microbiol. 2024 May 22;15:1398005. doi: 10.3389/fmicb.2024.1398005 (PMC11150647; doi:10.3389/fmicb.2024.1398005)

**Figure.S1.** Differential analysis of physicochemical characteristics.


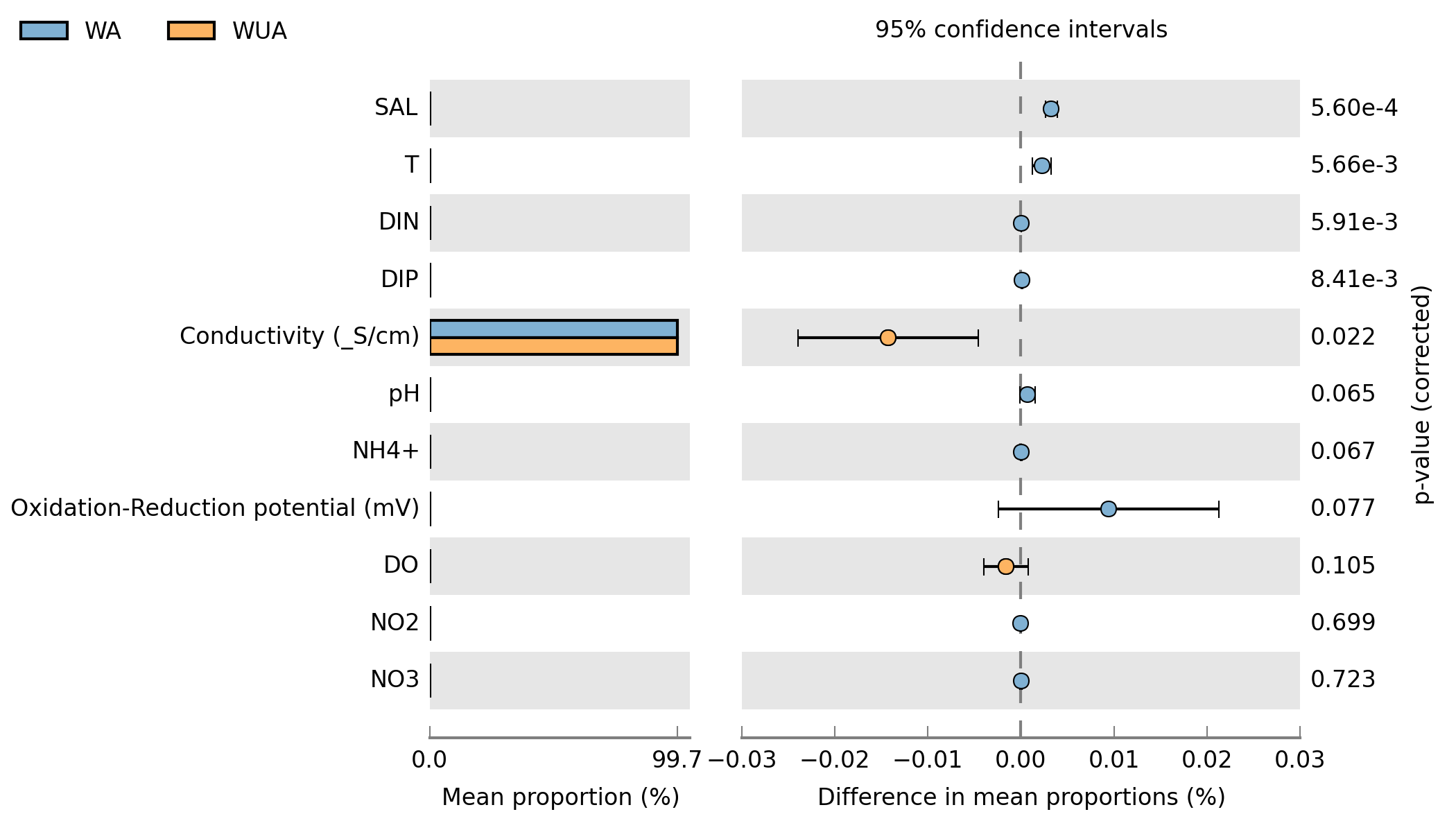


**Figure.S2.** Twenty dominant phylum are shown with their relative abundance; the remaining phyla are indicated as “others”. (a) Carbon metabolism (b) Nitrogen metabolism (c) Sulfur metabolism.


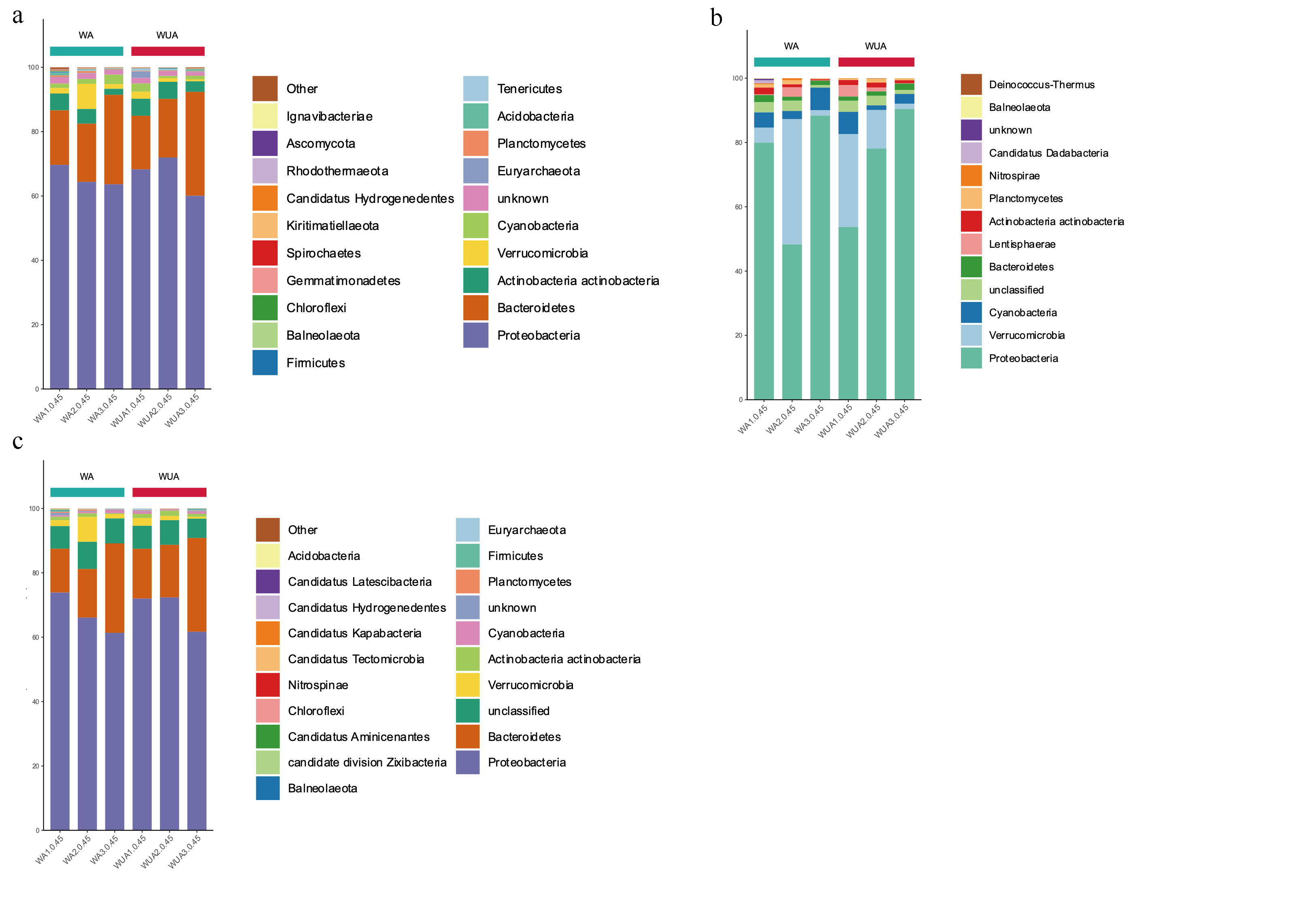


**Figure.S3.** Differential analysis of phosphorus metabolism-related genes.


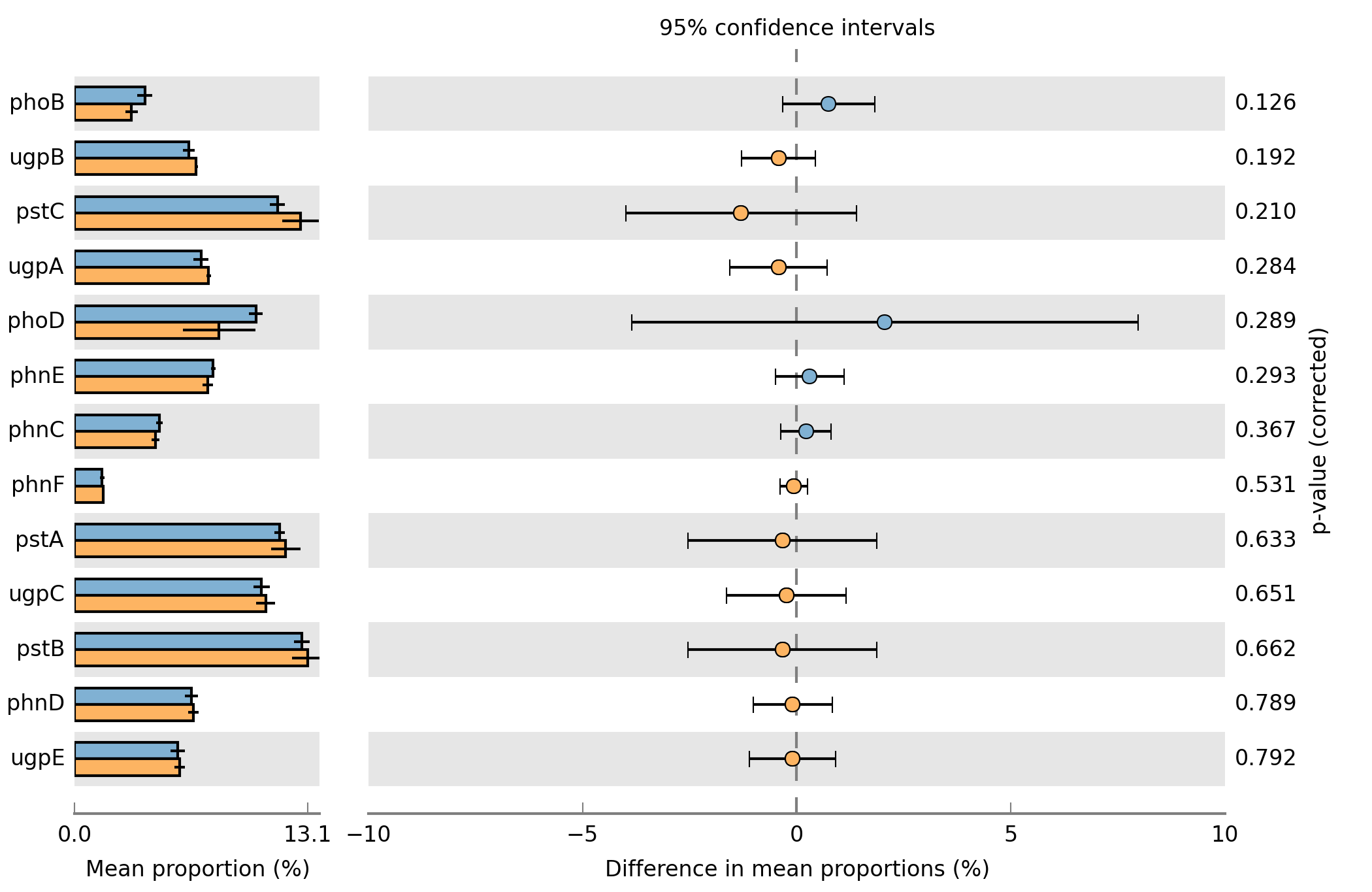


**Figure.S4.** Differential analysis of virulence genes.


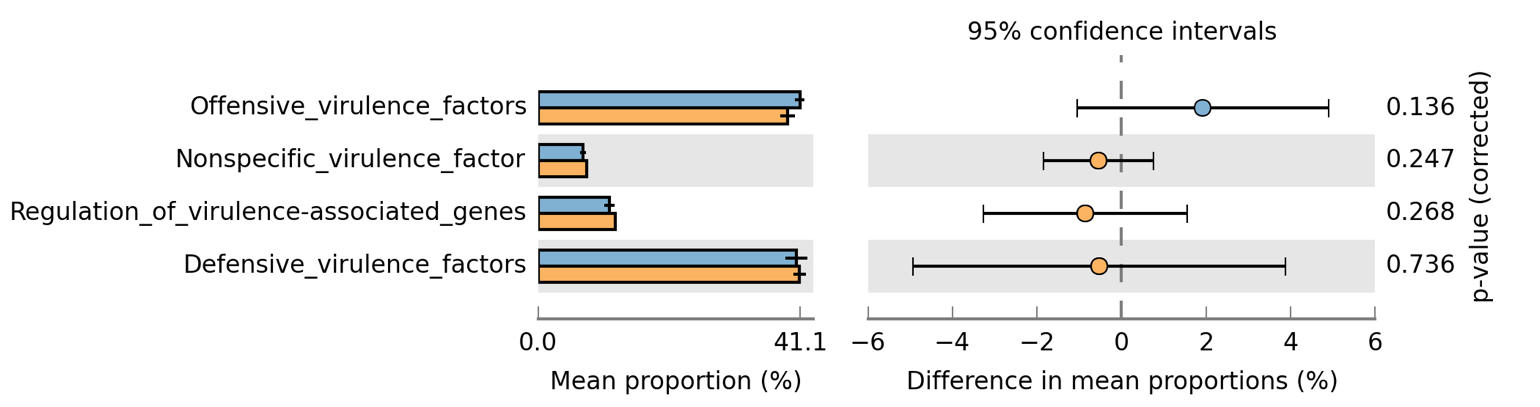


**Figure.S5.** Differential analysis of antimicrobial-resistant genes.


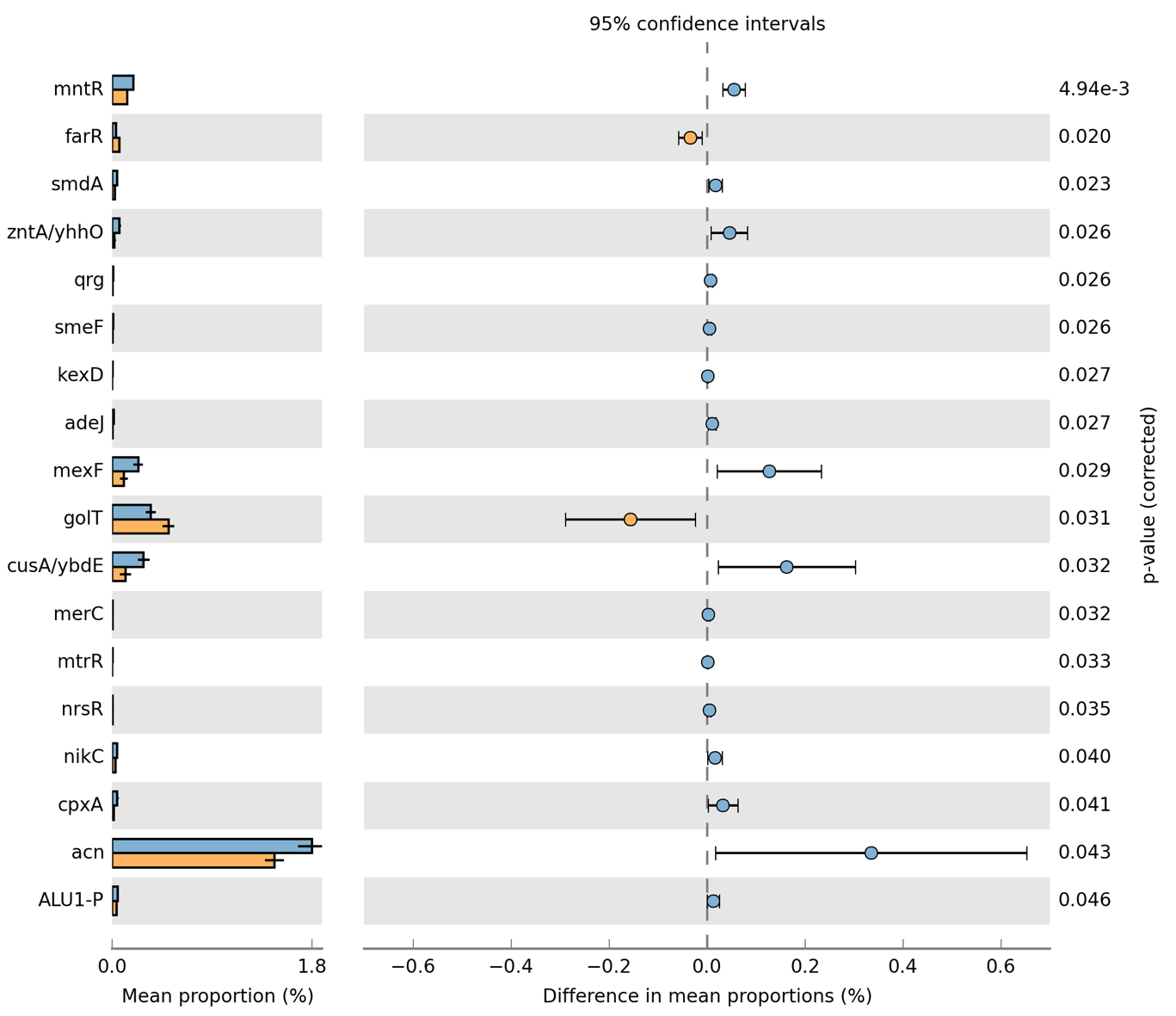

Supplement: Supplementary file 2 [file Data_Sheet_2.DOCX]
